# Metal Exchange in Thioguanosine Coordination Polymers of Gold (I) and Silver (I)

**DOI:** 10.1002/chem.202404318

**Published:** 2025-02-21

**Authors:** Chayanan Tangsombun, Liam F. McGarry, Osama El‐Zubir, Andrew Houlton, Benjamin R. Horrocks

**Affiliations:** ^1^ Chemical Nanoscience Laboratories School of Natural and Environmental Sciences Bedson Building Newcastle University Newcastle upon Tyne NE1 7RU UK; ^2^ Department of Chemistry University of York York YO10 5DD UK; ^3^ School of Chemistry University of Birmingham, Edgbaston Birmingham B15 2TT UK

## Abstract

Heterometallic coordination polymers of Au(I) and Ag(I) with 6‐thioguanosine, poly($\left[{\mathrm{Au}}_{x}{\mathrm{Ag}}_{1-x}(6-\mathrm{tG})\right]$ ), have been prepared and were observed to form hydrogels. We find that the composition of the heterometallic polymer is proportional to the mole fractions of the metals in the preparation solution. Optical absorption spectra show single peaks for λ>300 nm which can be interpolated in a linear manner between x =0.0 and x =1.0 consistent with the formation of a heterometallic polymer rather than a mixture of homopolymers. However photoluminescence and circular dichroism spectra are sensitive to the supramolecular structure of the polymers and show more complex behaviour. Atomic force microscopy indicated that the molecular chains of the Au homopolymer entwine to form strands that are predominantly right‐hand helices. The Ag homopolymer has previously been shown to form left‐hand helices. Intermediate compositions have more complex structures because of the competition between the left and right‐handed preferences of the homopolymers. Finally, we have shown that the metal‐ligand bonds are labile on a timescale of about 5 h at ambient temperature (about 293 K). Mixtures of homopolymers transform to the corresponding heterometallic coordination polymer by metal exchange as judged by optical absorption, photoluminescence and circular dichroism spectra.

## Graphical abstract

Gold(I) and silver (I) ions react with 6‐thioguanosine to form helical coordination polymers with metal‐sulfur chains. These polymers have a helical structure and show strong circular dichroism and photoluminescence. Mixtures of metal ions result in heterometallic coordination polymers. Further, mixtures of homopolymers will exchange metal ions over about 5 h to produce the corresponding heterometallic polymer at equilibrium.

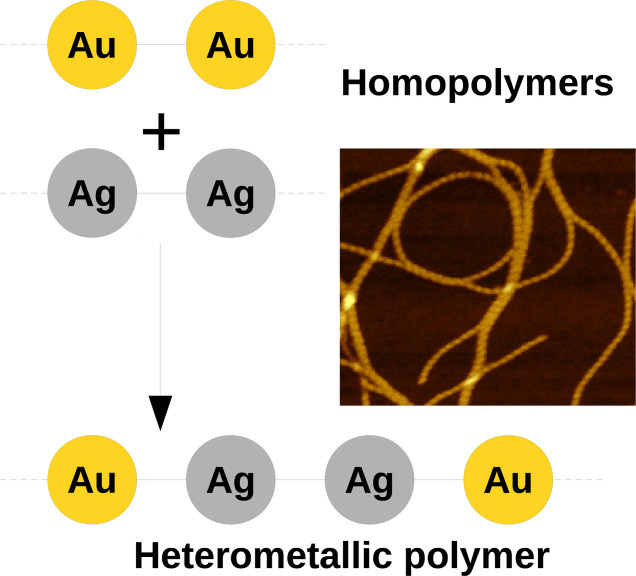

## 1 Introduction

Coordination polymers are an intensively‐investigated class of materials in which the polymer chain comprises coordination compounds.^[1]^ Coordination polymers may be crystalline,^[2]^ amorphous^[3]^ and may form gels.[^4^, ^5^] Part of the motivation for their study is the possibility to incorporate novel physico‐chemical properties, that are not readily available in organic polymers, via the choice of metal ion.[^6^, ^7^] Examples include catalysis,[^8^, ^9^, ^10^] sensing/anion binding,[^11^, ^12^, ^13^, ^14^] spectroscopic properties,[^15^, ^16^, ^17^] conductivity^[18]^ and magnetism.^[19]^

Heterometallic coordination polymers are an emerging class of molecular materials that offer the possibility to tune properties through controlled changes of the metal centres.[^20^, ^21^, ^22^, ^23^, ^24^, ^25^] Methods for the preparation of such materials are varied.^[26]^ For example, the 3D structures of metal‐organic frameworks, MOFs, have been shown to be sufficiently robust to allow post‐synthetic modification via heterometal‐exchange, generally by exposure of crystalline MOFs to solutions containing the heterometal ions.[^26^, ^27^]

For coordination polymers with lower dimensionality, especially 1D/chain structures, such a metal‐exchange approach has not been widely demonstrated. Instead, synthetic approaches to such compounds typically rely upon exploiting hard‐soft acid‐base combinations in one‐pot,^[28]^ using a metallo‐ligand approach[^26^, ^29^] or simple random doping using a mixture of different metal ions during the initial synthesis.

This last approach has been exploited recently to prepare Ag/Cu‐containing sheets of $[{\mathrm{Ag}}_{x}{\mathrm{Cu}}_{y}\left(p-{\mathrm{SPhCO}}_{2}H)\right]$ providing a means to tune the emission from green to red. Interestingly, heterometal exchange on $\left[\mathrm{Ag}(p-{\mathrm{SPhCO}}_{2}H)\right]$ with Cu(I) ions was ineffective, despite the Cu(I) analog having an isostructural, 2D, arrangement.^[30]^

**Figure 1:**
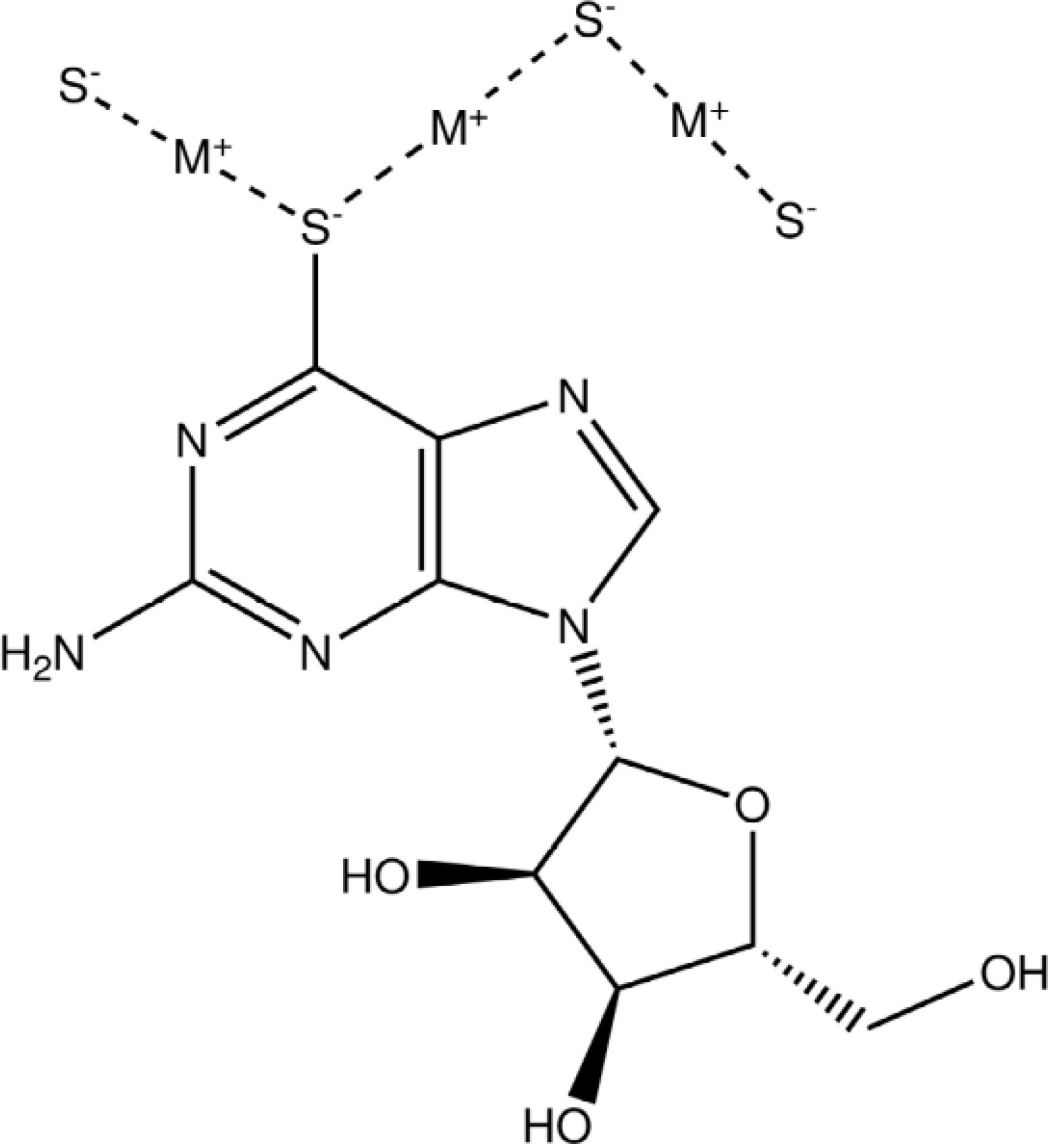
Schematic illustration of a poly([M(6‐tG)] chain where'M’ indicates either Ag(I) or Au(I). For the sake of clarity, only one (−)‐6‐thioguanosine group is shown in full. Throughout the text, poly($\left[{\mathrm{Au}}_{x}{\mathrm{Ag}}_{1-x}(6-\mathrm{tG})\right]$ ) indicates a coordination polymer where the mole fraction of Au(I) is denoted x.

We have been exploring coinage metal‐thiolate coordination polymers based on sulfur‐modified nucleosides[^31^, ^32^, ^33^, ^34^] and find that these materials often form hydrogels. In this work, we exploit this observation to demonstrate that both simple doping and heterometal exchange between polymers is possible in the 1D coordination polymer chains of gel‐forming coinage‐metal thiolates based on (−)‐6‐thioguanosine (6‐tGH). The basic structure of these polymers is illustrated in Figure 1. First, we use mass spectrometry and optical absorption spectroscopy to show that heterometallic coordination polymers rather than mixtures of homopolymers form from reactions of 6‐tGH with mixed aqueous solutions of Au(I) and Ag(I). Then we study the variation of the heterometallic polymer morphology and optical properties with composition, given by the fraction of Au in the preparation solution. Finally, we interpret the observation that the optical spectra of mixtures of homopolymers evolve towards the spectra of the corresponding heterometallic polymer as evidence for heterometal‐exchange in these systems.

## Experimental

### Materials

Reagents were obtained from Sigma‐Aldrich and used as‐received. Deionized water (Type 1, nominal 18.2 MΩ cm resistivity) was obtained from a Direct‐Q® 3 UV water purification train equipped with a reverse osmosis system (MerckMillipore).

### Synthesis of Ag(I)‐thioguanosine Coordination Homopolymer, Poly([Ag(6‐tG)])

${\mathrm{AgNO}}_{3}$ (2.7 mg, 16 μ mol) was dissolved in deionized water (0.6 mL), and the solution was then added to a dispersion of 6‐thioguanosine (6‐tGH, 4.8 mg, 16 μmol) in deionized water with a final combined volume of 1.6 mL. The concentration of all polymer samples in this report is described in terms of the ligand concentration and following this procedure it is 10 mM. The reaction mixture was stirred at room temperature for 5 min. The sample was then left for 2 h to ensure a pale yellow hydrogel formed. Gelation was tested by vial inversion. Rheological measurements are presented in the supporting information.

### Synthesis of Au(I)‐thioguanosine Coordination Homopolymer, Poly([Au(6‐tG)])

A suspension of 6‐thioguanosine (4.8 mg, 16 μ mol) in deionized water (1 mL) was sonicated and then stirred vigorously. One equivalent of Au(I) ion was added to this suspension to give a final volume of 1.6 mL. The Au(I) solution was produced by the reaction of ${\mathrm{HAuCl}}_{4}$ (5.5 mg, 16 μ mol) in 0.6 mL of water with two equivalents of 2,2‐thiodiethanol (4.0 mg, 3.2 μ L, 33 μ mol). The reaction mixture (10 mM by 6‐thioguanosine) was stirred at room temperature for 5 min, and left for 5 h to ensure the formation of an orange yellow hydrogel as verified by the simple vial inversion test. The gel contains the thiodiethanol‐derived oxidation product, ${\mathrm{HO}(\mathrm{CH}}_{2}{{)}_{2}{S=O(\mathrm{CH}}_{2})}_{2}\mathrm{OH}$ . Rheological measurements are presented in the supporting information.

### Synthesis of Ag(I)/Au(I)‐thioguanosine Heterometallic Coordination Polymer, Poly[Au_x_Ag_1‐x_(6‐tG)])

The various Au(I):Ag(I) ratios were reacted with 6‐thioguanosine in 1 : 1 overall metal:ligand stoichiometry and at a ligand concentration of 10 mM. To prepare a sample with x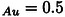 , the dispersion of thioguanosine (10 mg, 33 μ mol) in deionized water was stirred, followed by the addition of Au(I) ion (17 μ mol) generated from one equivalent of HAuCl_4_ with two equivalents of 2,2‐thiodiethanol. ${\mathrm{AgNO}}_{3}$ (17 μ mol) was immediately added to the reaction mixture (final combined volume 3.3 mL). The mixture was stirred for 5 min and formation of a yellow hydrogel was ensured by standing for 2–5 h and verified by a simple vial inversion test. Other values of x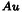 were obtained by adjusting the amounts of Au(I) and Ag(I) appropriately.

### Mixtures of Homopolymers

In order to investigate the possibility of metal exchange between polymers, some samples of mixtures of homopolymers were prepared. The homopolymers poly([Ag(6‐tG)]) and poly([Au(6‐tG)]) were prepared as described above and allowed to stand for about 40 min at which point poly([Au(6‐tG)]) is typically still a viscous liquid rather than a gel. The homopolymers were then mixed and their spectra were followed with time after mixing. In the case of circular dichroism studies, the sample was diluted to 0.2 mM immediately after mixing and subsequently the spectra were observed as a function of time.

We prepared different mixtures of polymers and, for comparison, different heterometallic coordination polymer preparations (in which metal ions were mixed before addition of ligand). In section 2.3 these are denoted M1‐M4 and described below.

M1 (heterometallic coordination polymer): equimolar quantities of Ag(I) and Au(I) ions were mixed first and added to suspensions of 6‐thioguanosine (10 mM) in water at an overall stoichiometry of 1 : 1 metal to ligand.

M2 (heterometallic coordination polymer): as M1, but the ligand was dissolved in a 20/80 by volume mixture of acetonitrile/water.

M3 (homopolymer mixture): poly([Ag(6‐tG)]) (2 mL, 10 mM) and poly([Au(6‐tG)]) (2 mL, 10 mM) were mixed at room temperature 40 minutes after their individual preparations.

M4 (homopolymer mixture): poly([Ag(6‐tG)]) (2 mL, 10 mM) and poly([Au(6‐tG)]) (2 mL, 10 mM) were mixed 40 minutes after their individual preparations and thereafter maintained at a temperature of 50 °C.

M5 (heterometallic coordination polymer): A half equivalent of Au(I) was added to 6‐thioguanosine in water (2 mL, 10 mM). After mixing, a half equivalent of ${\mathrm{AgNO}}_{3}$ was added to an overall stoichiometry of 1 : 1 metal to ligand.

### Rheology

The gelation of poly([Ag(6‐tG)]) and poly([Au(6‐tG)]) was studied using a Discovery Hybrid Rheometer HR‐2 (TA‐Instruments, DE, USA), equipped with a parallel plate geometry (20 mm diameter). The frequency and temperature were set constant at 1 Hz and 22 °C, respectively to study real‐time hydrogel formation. The initial solutions of poly([Ag(6‐tG)]) and poly([Au(6‐tG)]) at 10 mM were prepared, and the solutions (400 μ L) were loaded on the plate. The gap between the upper and lower plate was set at 1100 μ m. The preparation and loading procedures took 120 s after which the gel formation was observed as a function of time. Subsequently scans of (i) frequency over the range from 0.1 rad s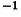 to 100 rad s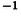 ; (ii) amplitude (torque) from about 0.01 μ N to about 550 μ N and (iii) shear rate from 1 s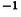 to 100 s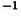 were recorded.

### Energy Dispersive Analysis of X‐rays

Samples of coordination polymers (10 mM) were precipitated with acetone. The precipitate was washed twice with acetone/water to remove unreacted reagents and then dried in a vacuum oven. Finally, the heterometallic polymers were dispersed in water, deposited on holey carbon (3 μ L) and again dried in the vacuum oven. The samples were imaged by scanning electron microscopy (SEM) with energy dispersive X‐ray (EDX) analysis using a JSM‐5600LV instrument (Jeol) at 20 kV and 20 mm working distance. The values plotted are the mean and standard deviation of 5 measurements that were taken on different areas of each sample.

### Mass Spectrometry

The mass spectrometer used was a Xevo G2‐XS quadrupole time‐of flight (QToF) MS with an electrospray ionisation (ESI) source (Waters). The mass spectrum was collected in positive ion mode. The poly($\left[{\mathrm{Au}}_{0.5}{\mathrm{Ag}}_{0.5}\left(6-\mathrm{tG}\right)\right]$ ) sample concentration was 500 μ M and was obtained by 20‐fold dilution of a 10 mM preparation with deionized water.

### Atomic Force Microscopy

Silicon wafers, (100) oriented, were cut into chips of about 1×1 cm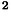 , cleaned, washed with acetone, and dried with $N_{2}$ gas. Samples of polymer prepared at 10 mM were diluted to 10 μ M with water. 1 μ L aliquots of polymer solution were pipetted onto a clean Si chip and the sample was dried in air. Atomic force microscopy (AFM) images were acquired on a Bruker MultiMode 8 instrument using ScanAsyst in Air mode. Silicon tips on silicon nitride cantilevers (ScanAsyst, Bruker) were used for imaging. The nominal tip radius was approximately 2 nm, the resonant frequency was 150 kHz, and the spring constant was $k≃0.7\;{\mathrm{nm}}^{-1}$ . The image data was analyzed using Gwyddion 2.41 software (http://gwyddion.net/).^[35]^ An isolation table enclosure was used to reduce vibrational noise from the surroundings.

### UV‐Vis Absorption Spectroscopy

UV‐vis spectra of poly([Au(6‐tG)]), poly([Ag(6‐tG)]) and poly($\left[{\mathrm{Au}}_{x}{\mathrm{Ag}}_{1-x}\left(6-\mathrm{tG}\right)\right]$ ) heterometallic coordination polymers at a concentration of 10 mM in terms of 6‐tG were measured using the pedestal mode (min. pathlength 0.03 mm) of a NanoDrop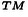 One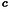 spectrophotometer (ThermoFisher Scientific) at room temperature. The blank was 3 μ L of deionized water.

To study the evolution of UV‐Vis spectra with time, 10 mM of the polymer was diluted to 0.1 mM and UV‐vis spectra were recorded using the cuvette mode (10 mm pathlength) of the Nanodrop at room temperature (about 293 K).

### Circular Dichroism Spectroscopy

CD spectra were recorded on a J‐810 spectropolarimeter (Jasco). Scan speed was set to 100 nm min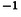 and the response time to 2 s. The data was collected from 500 nm to 190 nm and 2 scans were accumulated and averaged. Deionized water was run as a blank for baseline correction. Poly([Au(6‐tG)]), poly([Ag(6‐tG)]) and poly($\left[{\mathrm{Au}}_{x}{\mathrm{Ag}}_{1-x}\left(6-\mathrm{tG}\right)\right]$ ) at concentration a of 0.2 mM were prepared by dilution of samples originally prepared at 10 mM. CD spectra of each polymer were immediately recorded after dilution in the quartz cuvette of 10 mm pathlength. CD spectra of 6‐thioguanosine (1 mg/16.7 mL of water) were also recorded in the quartz cuvette with the same pathlength. Mixtures M1 to M5 described above were also diluted to 0.2 mM to record CD spectra.

### Luminescence Spectroscopy

The excitation and emission maps (EEM) of the luminescence spectra of poly([Au(6‐tG)]), poly([Ag(6‐tG)]), and poly($\left[{\mathrm{Au}}_{x}{\mathrm{Ag}}_{1-x}\left(6-\mathrm{tG}\right)\right]$ ) heterometallic coordination polymers at 10 mM were measured using a quartz cuvette of 10 mm pathlength on a Shimadzu RF‐6000 fluorescence spectrometer. The excitation wavelength was recorded over the range of 300–500 nm and the emission was scanned over range of 400–800 nm. The slit widths of both excitation and emission monochromators were set to be 5 nm. The scan speed was set at 600 nm min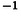 .

Individual emission spectra of 10 mM samples of homopolymers and heterometallic coordination polymers ($\lambda_{exc}=420$ nm) were measured on a SPEX Fluoromax spectrofluorometer. The time‐evolution of the spectra over a period of hours were also recorded on the SPEX Fluoromax spectrofluorometer at room temperature (about 293 K).

## 2 Results and Discussion

We study the preparation of heterometallic coordination polymers with the monomer units being complexes of either Au(I) or Ag(I) with the ligand 6‐thioguanosine (6‐tGH). These polymers are denoted poly($\left[{\mathrm{Ag}}_{1-x}{\mathrm{Au}}_{x}\left(6-\mathrm{tG}\right)\right]$ ) where x is the mole fraction of Au. Both homopolymers (x=0,x=1 ) and the heterometallic polymers (0<x<1 ) form hydrogels. The heterometallic polymers are prepared by the reaction of the ligand with mixed aqueous solutions of Au(I) and Ag(I) salts in various ratios. First, we demonstrate that this strategy does indeed produce heterometallic polymers rather than mixtures of homopolymers using mass spectrometry, optical spectroscopy and energy‐dispersive X‐ray spectroscopy (EDX). Next, we discuss the morphology of the heterometallic polymers as observed in the xerogel form by atomic force microscopy (AFM). Finally, we demonstrate that these systems are labile in the sense that mixing samples of homopolymers leads to the formation of heterometallic polymers on a timescale of about 5 h and that the mixed system appears to be under thermodynamic control.

### 2.1 Evidence for Heterometallic Coordination Polymer Formation

#### 2.1.1 Mass Spectrometry

The positive ion mode mass spectra (supporting information) of poly($\left[{\mathrm{Au}}_{0.5}{\mathrm{Ag}}_{0.5}\left(6-\mathrm{tG}\right)\right]$ ) samples indicated the presence of the following species: ${\left[\mathrm{Ag}{\left(6-\mathrm{tGH}\right)}_{2}\right]}^{+}$ (m/z: Calc 705.04, 707.04; Found 705.049, 707.049); ${\left[\mathrm{Au}{\left(6-\mathrm{tGH}\right)}_{2}\right]}^{+}$ (m/z: Calc 795.10; Found 795.115); [AgAu(6‐tG)(6‐tGH)]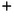 (m/z: Calc 901.00, 903.00; Found 901.00, 903.00). The m/z values quoted include the major isotopes of the ${\mathrm{Ag}}^{+}$ ions: 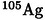 (50 %) and 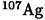 (46 %). In principle, the ${\left[\mathrm{Ag}{\left(6-\mathrm{tGH}\right)}_{2}\right]}^{+}$ and ${\left[\mathrm{Au}{\left(6-\mathrm{tGH}\right)}_{2}\right]}^{+}$ fragments could derive from either monometallic polymers or a bimetallic coordination polymer. However, the [AgAu(6‐tG)(6‐tGH)]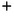 ion in the mass spectrum of x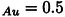 samples confirmed the formation of heterometallic coordination polymers.

#### 2.1.2 Optical Spectroscopy of Heterometallic Coordination Polymers

**Figure 2:**
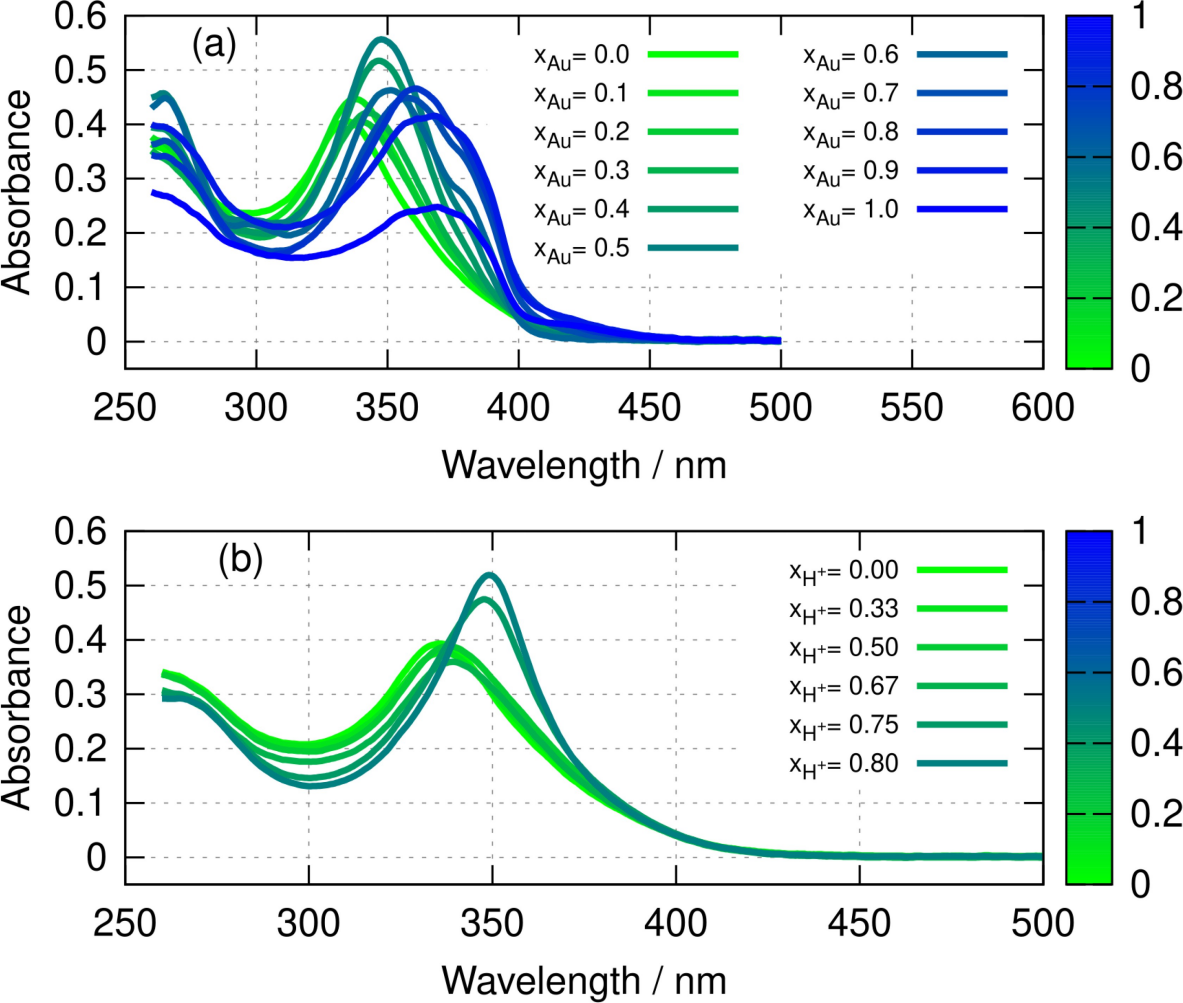
(a) Absorption spectra of poly(${[\mathrm{Ag}}_{1-x}{\mathrm{Au}}_{x}\left(6-\mathrm{tG}\right)]$ ). The colour bar indicates the mole fraction, x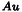 of gold in the preparation solution. (b) Absorption spectra of poly([Ag(6‐tG)]) upon addition of HCl(aq). The colour bar indicates the mole fraction of $H^{+}\left(\mathrm{aq}\right)$ , $x_{H^{+}}$ in the solution compared to Ag in the polymer. The concentration of the polymers was 10 mM in terms of 6‐tG in both cases.

The optical absorption spectra of poly($\left[{\mathrm{Ag}}_{1-x}{\mathrm{Au}}_{x}\left(6-\mathrm{tG}\right)\right]$ ) vary with the mole fraction x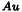 in the preparation solution. The main peak in the near UV occurs at $\lambda_{max}=334.5$ nm for the Ag homopolymer and at $\lambda_{max}=369.0$ nm for the Au homopolymer. There are two limiting cases for the variation of the spectra with x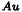 : (i) heterometallic coordination polymer formation with a single $\lambda_{max}$ that may be estimated by linear interpolation between the values for the homopolymers and (ii) formation of a mixture of homopolymers in which the spectra are a weighted sum of the homopolymer spectra. Figure 2a shows the experimental spectra, which are broadly consistent with case (i). Note that the spectra for x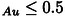 have a $\lambda_{max}$ that is intermediate between the values for the homopolymers and with unchanged peak width, which is inconsistent with case (ii). The spectrum for x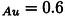 does show a clear shoulder on the long wavelength side of the peak, but this is not evident for higher mole fractions. Overall, the data is best represented by case (i) – heterometallic coordination polymer formation.

However, the possibility of protonation effects on the $\lambda_{max}$ must be considered. Figure 2b shows that addition of a strong acid also results in a shift of $\lambda_{max}$ to longer wavelengths for poly([Ag(6‐tG)]). This is relevant because, as the mole fraction of ${\mathrm{HAuCl}4}_{4}$ increases, the acidity of the solution increases and therefore we have tested the extent to which the absorption maximum shift might reflect protonation of the thioguanosine in poly($\left[{\mathrm{Au}}_{x}{\mathrm{Ag}}_{1-x}(6-\mathrm{tG})\right]$ ).

A simple model for the effect of protonation can be constructed under the assumption of a single p$K_{a}$ for the polymer and a peak wavelength that shifts in proportion to the extent of protonation ([B] versus $\left[BH^{+}\right]$ ) where B and $BH^{+}$ represent deprotonated and protonated forms of monomer units within the polymer. Experimentally, we control the ratio r=F/M where F is the formal concentration of added protons as HCl(eq) and M is the total metal concentration, also equal to the total monomer unit concentration. In order to display the results on a single graph for comparison, we plot the wavelength of maximum absorption against a proton mole fraction x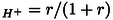 . Calculation details are given in the supporting information.

**Figure 3:**
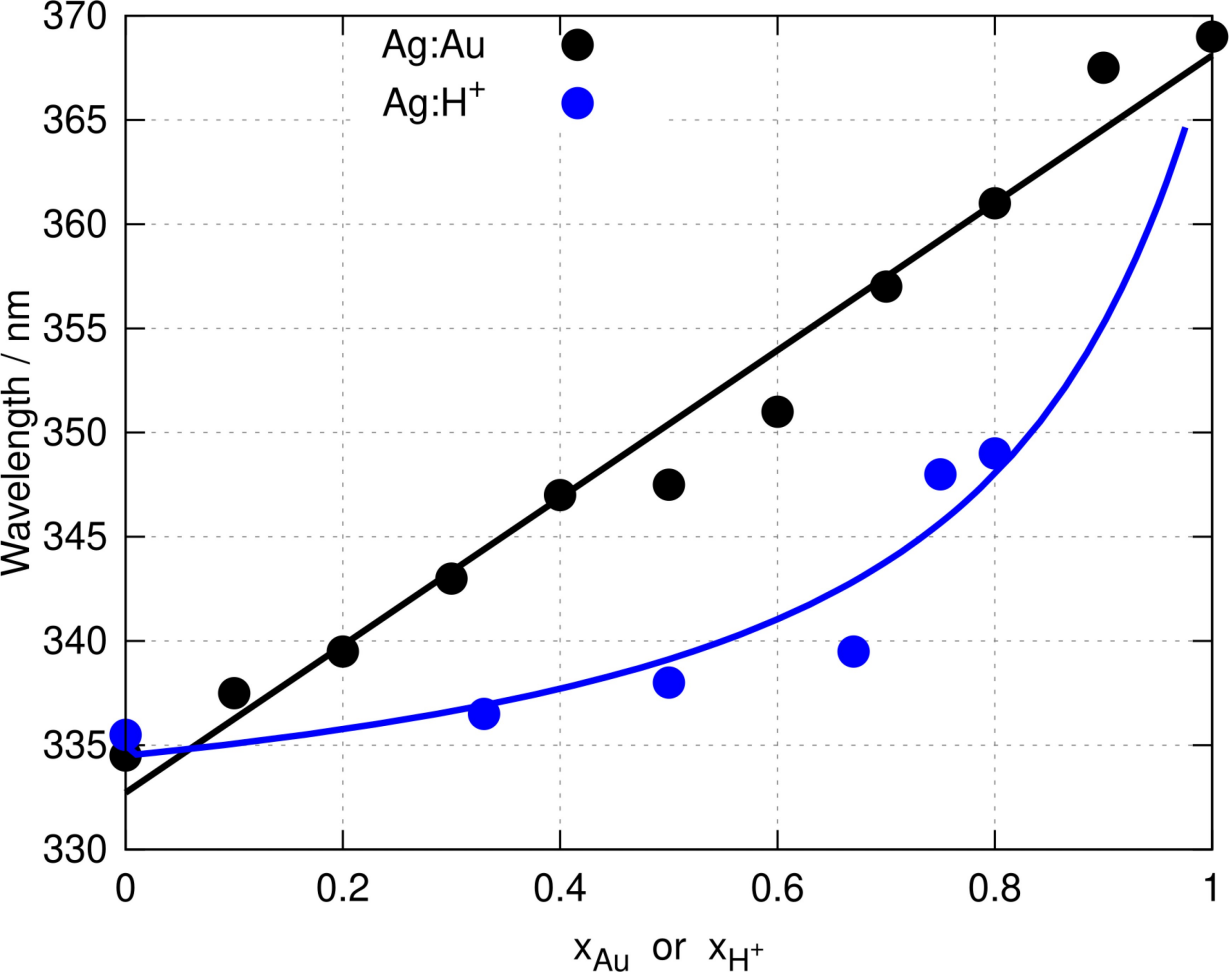
Wavelength of the absorption maxima of poly($\left[{\mathrm{Ag}}_{1-x}{\mathrm{Au}}_{x}\left(6-\mathrm{tG}\right)\right]$ ) (black) and of poly([Ag(6‐tG)]) upon addition of HCl(aq) (blue) against mole fraction of Au or H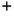 in the preparation solution. The symbols show the experimental data as a function of the mole fraction of gold x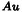 (black) or of $x_{H^{+}}$ (blue). A regression line (black) is shown for $\lambda_{max}$ on x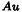 and an acid/base equilibrium was the regression model for $\lambda_{max}$ on x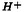 (blue).

This model is shown as a blue line in Figure 3 which is a least‐squares fit to the experimental data extracted from Figure 2b. The experimental data are shown as blue symbols. It is clear that the effect of addition of $H^{+}$ to poly([Ag(6‐tG)]) is different from the effect of increasing the mole fraction of Au in poly(${[\mathrm{Ag}}_{1-x}{\mathrm{Au}}_{x}(6-\mathrm{tG})]$ ). Replacement of Ag(I) by Au(I) produces a linear variation of $\lambda_{max}$ consistent with our interpretation of heterometallic coordination polymer formation. Addition of $H^{+}$ produces a smaller, non‐linear change in $\lambda_{max}$ and it is worth noting that at x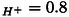 the ratio of acid : metal is 4 : 1, which is much higher than the 1 : 1 ratio that would be obtained by complete replacement of ${\mathrm{AgNO}}_{3}$ by Au(I) in the heterometallic coordination polymer synthesis. We conclude that protonation of the monomer units is not responsible for the shift in $\lambda_{max}$ observed in Figure 2a.

#### 2.1.3 Energy‐Dispersive Analysis of X‐rays

**Figure 4:**
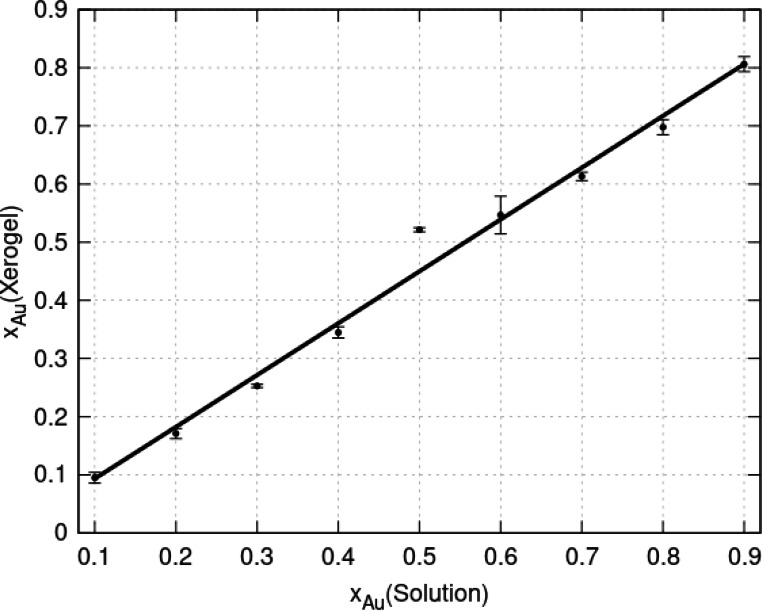
Mole fraction of Au in the xerogel of poly($\left[{\mathrm{Au}}_{x}{\mathrm{Ag}}_{1-x}(6-\mathrm{tG})\right]$ ) determined by EDX against the mole fraction of Au in the preparation solution. The error bars represent standard deviations of the 5 measurements taken for each mole fraction.

The mole fraction of Au in poly($\left[{\mathrm{Au}}_{x}{\mathrm{Ag}}_{1-x}(6-\mathrm{tG})\right]$ ) will be determined by the composition of the solution, kinetic and thermodynamic factors. Under kinetic control, different rates of polymerisation of Au(I) and Ag(I) would produce heterometallic coordination polymers enriched in the metal with more rapid polymerization kinetics. In a system under thermodynamic control, the composition of lowest overall free energy is favoured. The actual composition of the xerogel can be measured simply using energy dispersive analysis of X‐rays on a dry sample under the beam of an electron microscope. Figure 4 shows a plot of Au mole fraction in such a xerogel against Au mole fraction in the preparation solution. The coefficient of determination is $r^{2}=0.98$ and the slope of the regression line is 0.89±0.017 .^1^ This indicates a small preference for Ag over Au in the polymer, but the difference from equality of x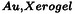 and x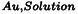 is so small that we cannot rule out an effect of the washing protocol on the composition of the xerogel. The intercept is $\left(4.3\pm 9.8\right)\times 10^{-3}$ , which is zero within the precision of the measurement. The composition of the polymer is therefore clearly proportional to the composition of the reaction solution over this range. Unless otherwise stated, we will continue to use $x_{\mathrm{Au}}$ to refer to the mole fraction of Au in the preparation solution in the discussions that follow.

### 2.2 Variation of Heterometallic Coordination Polymer Morphology and Properties with Composition

#### 2.2.1 Photoluminescence and Circular Dichroism

**Figure 5:**
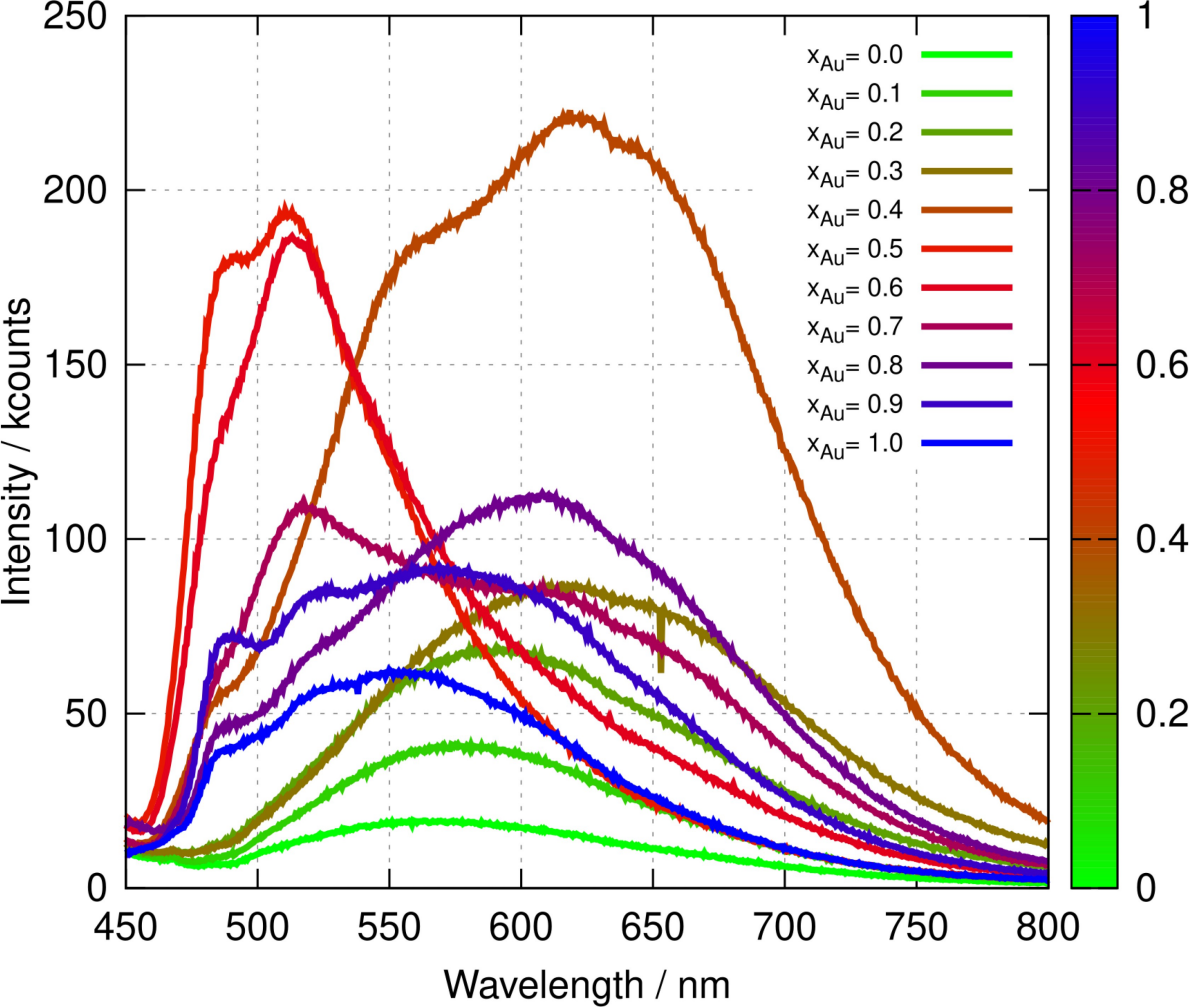
Photoluminescence spectra of poly($\left[{\mathrm{Au}}_{x}{\mathrm{Ag}}_{1-x}(6-\mathrm{tG})\right]$ ). The excitation wavelength was $\lambda_{exc}=420$ nm and the colour bar indicates the mole fraction, x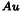 of gold in the preparation solution. The concentration of the polymers was 10 mM in terms of 6‐tG in both cases.

**Figure 6:**
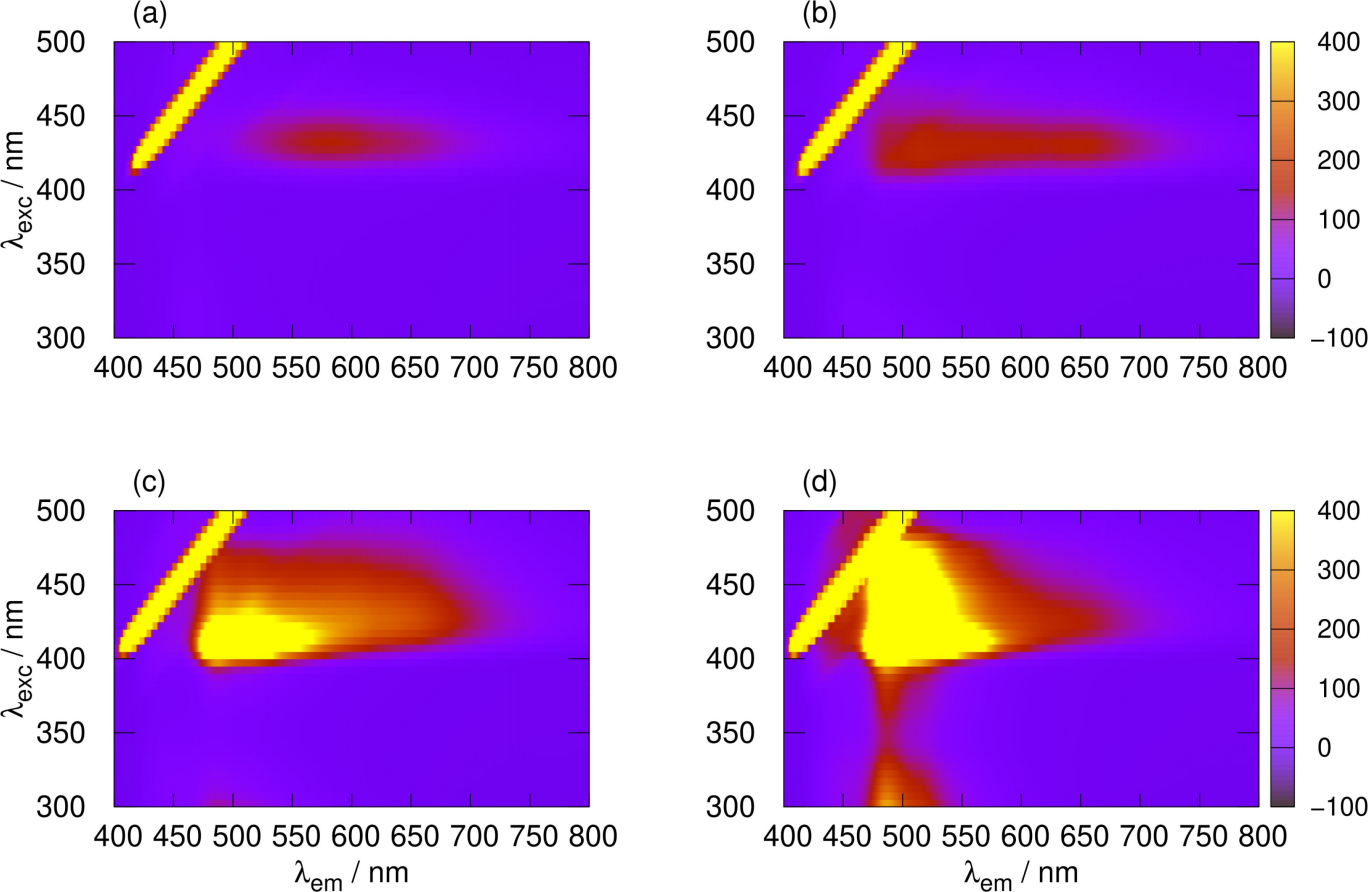
Luminescence excitation‐emission maps for a selection of heterometallic coordination polymers of composition poly($\left[{\mathrm{Au}}_{x}{\mathrm{Ag}}_{1-x}(6-\mathrm{tG})\right]$ ) and concentration 10 mM in terms of 6‐tG. (a) x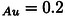 ; (b) x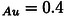 ; (c) x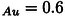 ; and (d) x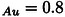 . The y‐axis gives the excitation wavelength, the x‐axis gives the emission wavelength and the colour scale represents the emission intensity in kcounts.

Figure 5 presents the photoluminescence spectra of poly($\left[{\mathrm{Au}}_{x}{\mathrm{Ag}}_{1-x}(6-\mathrm{tG})\right]$ ) as a function of the mole fraction of Au in the preparation solution. Unlike the absorption spectra, the photoluminescence spectra are not well‐described as linear interpolations between the spectra of the homopolymers. For example, the spectra for x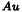 =0.5 and 0.6 show emission maxima that are shifted to higher energy compared to the two homopolymers. At low x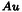 , the spectra resemble those of poly([Ag(6‐tG)]) with a broad, featureless band and a $\lambda_{max}$ in the range 570–620 nm as previously reported for the homopolymer.^[32]^ Up to about x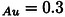 , the intensity increases smoothly with a red‐shift in the band position, but little change in the overall shape of the spectrum. These spectra can be assigned to a ligand‐to‐metal charge transfer (LMCT) transition.[^34^, ^36^] At x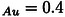 , the intensity is much larger than at x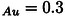 and some structure is evident in the band‐shape. For x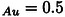 and x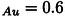 , the band sharpens, blue‐shifts to give $\lambda_{max}≃511$ nm and shows the vibronic structure typical of a ligand‐centred (LC) transition as observed in other Au(I) complexes,[^37^, ^38^] including $\mathrm{Au}\left(6-\mathrm{tGH}\right)2^{+}$ .^[34]^ Above x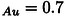 , the vibronic structure is less conspicuous and the spectrum tends towards a broad band typical of a LMCT. Excitation‐emission maps (Figure 6) display the changing nature of the electronic transitions with x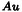 . The vibronic structure at x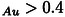 and the blue‐shift in the emission are clear in Figure 6c,d. There is also evidence of a second transition at λ≃300 for x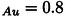 in Figure 6d.

A comparison of Figure 2 and 3 shows that the Stokes shift varies strongly with composition. At x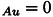 , the Stokes shift is about Δλ=240 nm and at x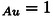 it is about 190 nm. Large Stokes shifts are typical for LMCT transitions which are strongly affected by solvation. Further, the involvement of a triplet 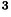 LMCT state owing to enhanced spin‐orbit coupling in the presence of the metal ions is likely. In contrast the shifts for x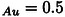 and 0.6 are about 160 nm measured to the emission maximum near 510 nm and 140 nm measured to the peak near 490 nm. These shifts are consistent with a triplet 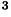 LC transition.

**Figure 7:**
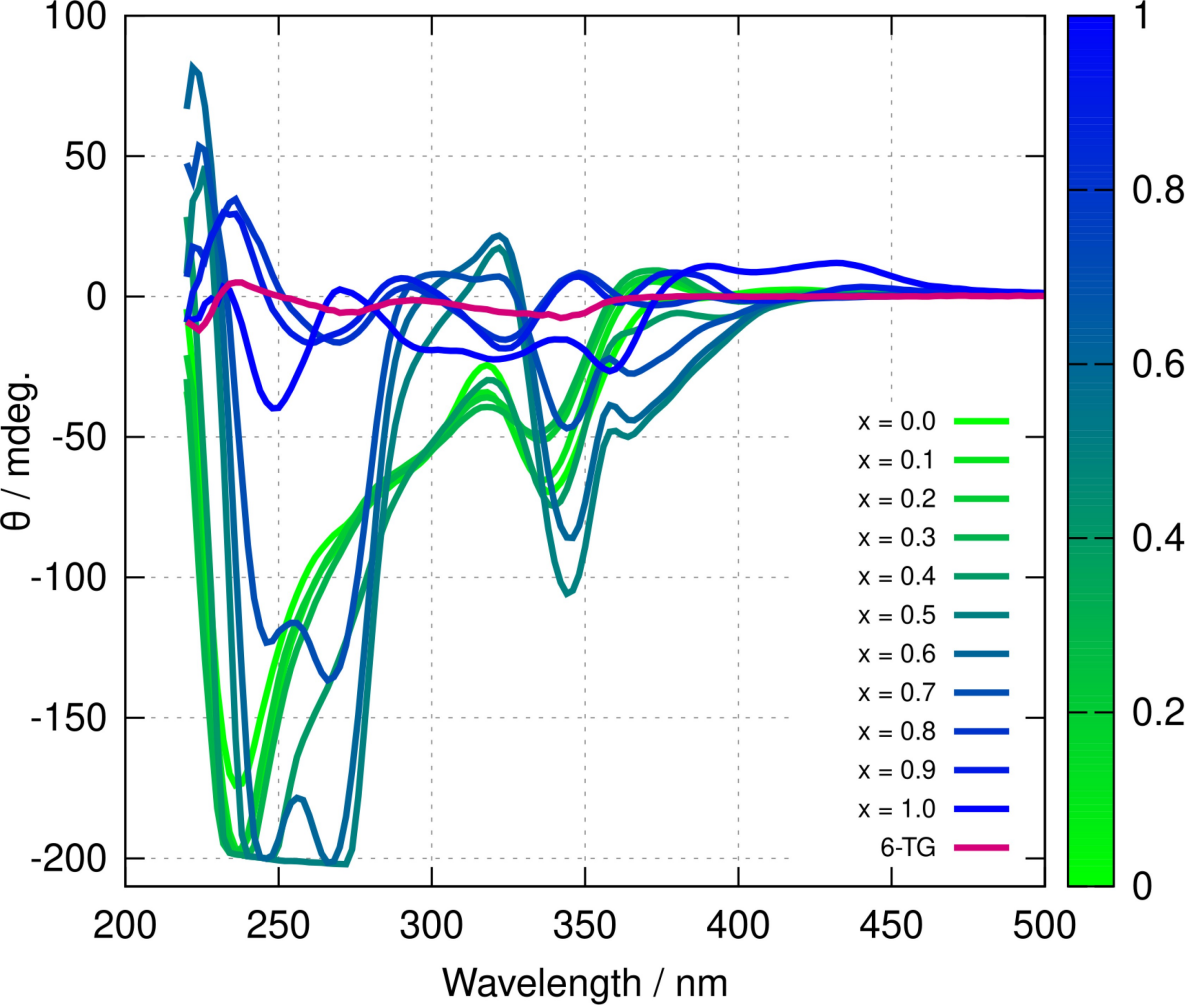
Circular dichroism spectra of poly($\left[{\mathrm{Ag}}_{1-x}{\mathrm{Au}}_{x}\left(6-\mathrm{tG}\right)\right]$ ). The colour bar indicates the mole fraction, x of gold. The concentration of the polymers was 0.2 mM in terms of 6‐tG. The red line shows the CD spectrum of the 6‐tG monomer in aqueous solution.

The circular dichroism (CD) spectra also show a complex behaviour as a function of x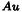 (Figure 7). Both homopolymers and all the heterometallic coordination polymers show enhanced CD compared to the ligand; this is evidence of some helical supramolecular structure in the polymers as has previously been shown.[^31^, ^32^] At low x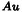 the CD spectrum resembles that of poly([Ag(6‐tG)]) with two main negative bands, one at λ=340 nm at the same position as the transition in the ligand associated with the thionate moiety, and the second, intense, band near 240 nm.^[32]^ In contrast, the poly([Au(6‐tG)]) spectrum shows a band near 420 nm associated with the Au−S chain.^[31]^ At intermediate values of x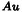 there is first a gradual enhancement and red‐shift of the negative peaks at 240 nm and 340 nm. At x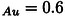 a new peak (266 nm) is visible, although it should be noted that the detector is saturated in this region for the x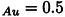 sample which shows a broad band that may also contain this new transition. Above x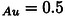 the CD signal decreases strongly and at x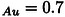 the sign of the feature at 340 nm changes to positive. These changes strongly suggest that the handedness of the supramolecular structure changes as the sample composition evolves and this motivated a study by atomic force microscopy.

#### 2.2.2 Atomic Force Microscopy

**Figure 8:**
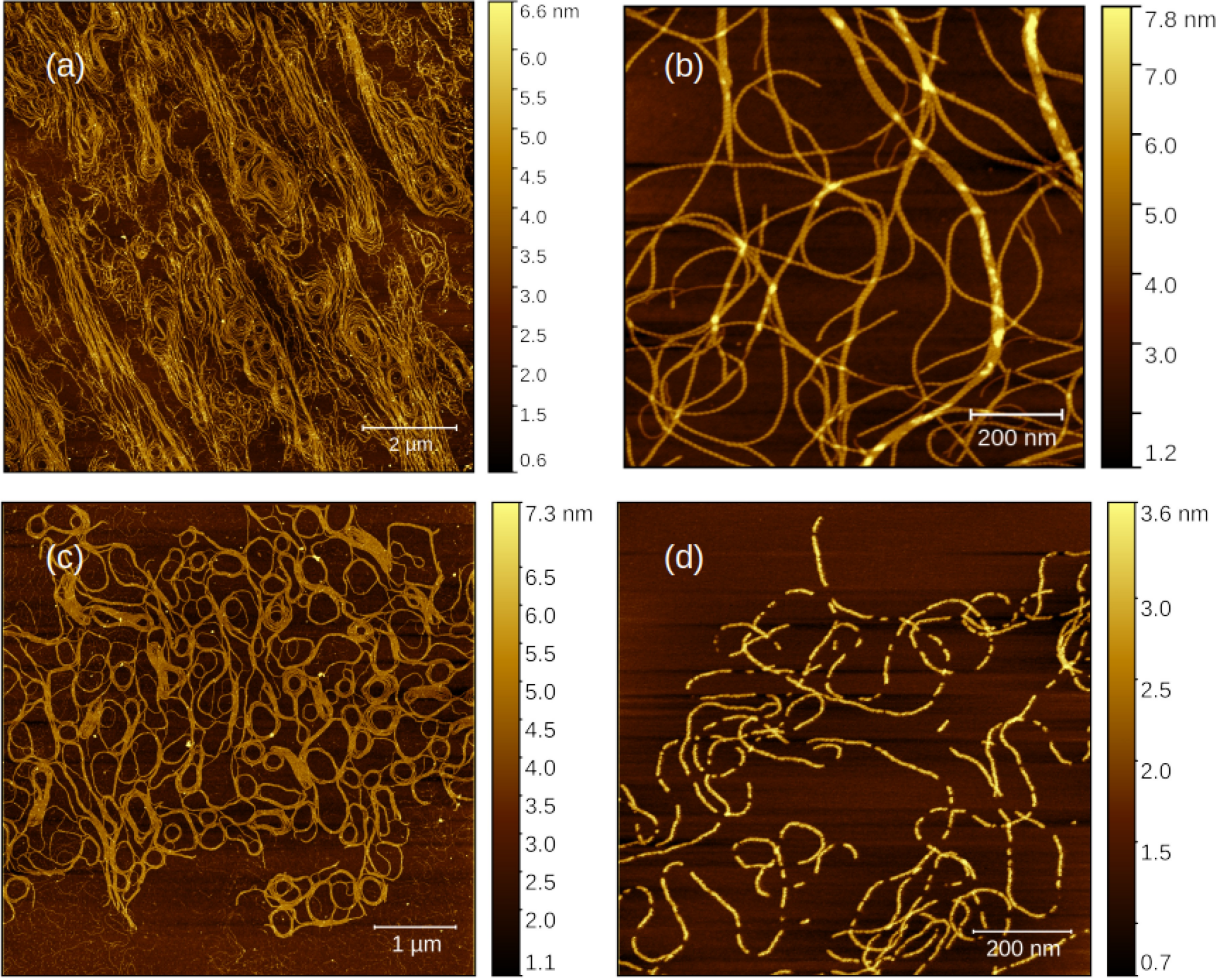
AFM images of the Au and Ag homopolymers in air at low (a, c) and higher (b, d) resolution. (a, b) poly([Au(6‐tG)]) and (c, d) poly([Ag(6‐tG)]). The colour scale indicates the height / nm.

The morphology of the polymer xerogels was studied by dilution of the 10 mM gel to 10 μ M, drop‐casting onto a silicon wafer, drying in air and then imaging by atomic force microscopy. Figure 8 displays images of the homopolymers poly([Au(6‐tG)]) and poly([Ag(6‐tG)]) on different length scales. On the larger length scale (Figure 8a,c) the two homopolymers both show long strands, but the poly([Ag(6‐tG)]) strands have a greater tendency to form loops and the poly([Au(6‐tG)]) strands a greater tendency to be linear. This suggests poly([Au(6‐tG)]) strands are stiffer and this is supported by the short length scale images (Figure 8b,d). Analysis of the height profiles across the strands (supporting information) confirms that poly([Au(6‐tG)]) strands are thicker (Table 1) and are generally unbroken in contrast to the poly([Ag(6‐tG)]) strands. These observations are consistent with the rheology data (supporting information) which show that the poly([Au(6‐tG)]) gels have a higher storage modulus than the poly([Ag(6‐tG)]) gels.

**Table 1:** Heights of strands from AFM image profiles. The uncertainties quoted are standard errors.

Polymer	Height/nm
poly([Au(6‐tG)])	2.61±0.044
poly([Ag(6‐tG)])	1.46±0.023
poly($\left[{\mathrm{Au}}_{0.5}{\mathrm{Ag}}_{0.5}\left(6-\mathrm{tG}\right)\right]$ )	1.61±0.018
poly(${[\mathrm{Au}}_{0.2}{\mathrm{Ag}}_{0.8}\left(6-\mathrm{tG}\right)]$ )	1.63±0.024

We have previously reported high resolution AFM images of poly([Ag(6‐tG)]) and found the strands to be left‐handed helices with a pitch of 11 nm.^[32]^ Surprisingly, the poly([Au(6‐tG)]) strands in Figure 8b are predominantly right‐handed. This is clearly visible in a zoomed‐in region of the image (supporting information). Although the interactions responsible for the preferred handedness of the helices are hard to establish, this finding is consistent with the change in sign of the CD signal for the thionate band at λ=340 nm in Figure 7 as the mole fraction x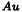 exceeds 0.7.

**Figure 9:**
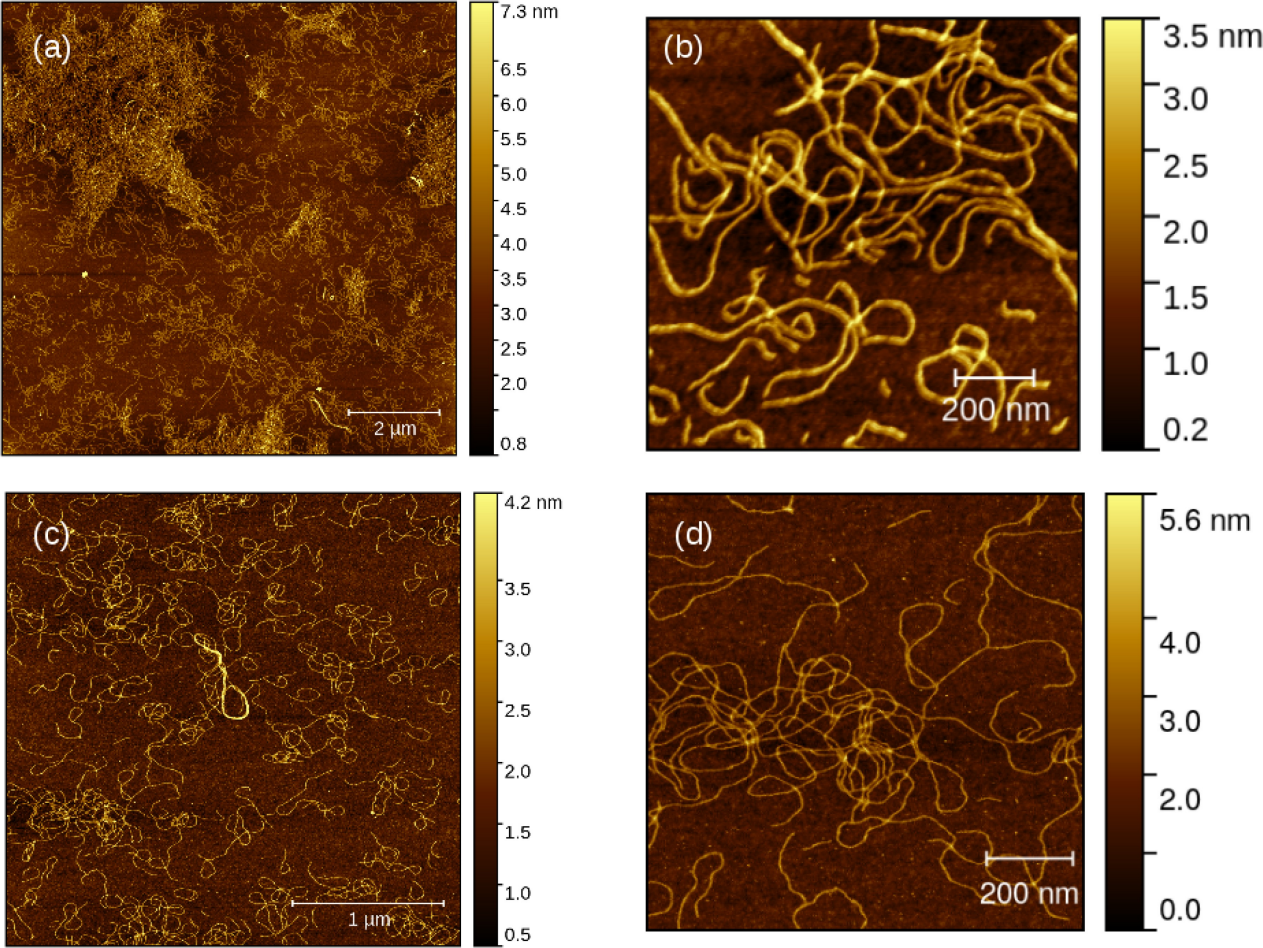
AFM images of two Au/Ag heterometallic coordination polymers . (a,b) poly($\left[{\mathrm{Au}}_{0.5}{\mathrm{Ag}}_{0.5}\left(6-\mathrm{tG}\right)\right]$ ) and (c,d) poly($\left[{\mathrm{Au}}_{0.2}{\mathrm{Ag}}_{0.8}\left(6-\mathrm{tG}\right)\right]$ ). The colour scale indicates the height / nm.

**Figure 10:**
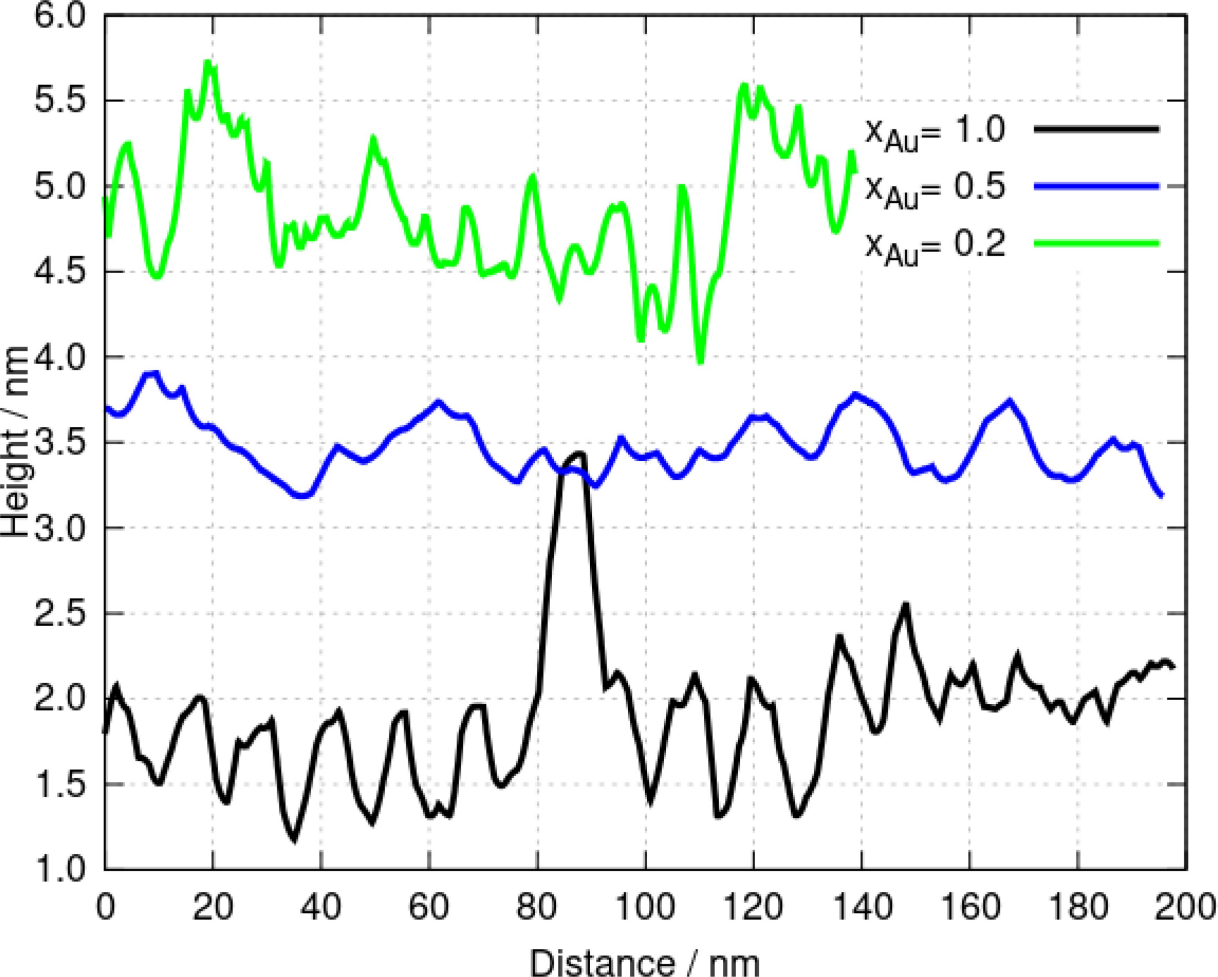
AFM profiles along strands of poly([Au(6‐tG)]) and poly($\left[{\mathrm{Au}}_{x}{\mathrm{Ag}}_{1-x}(6-\mathrm{tG})\right]$ ) for x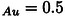 and x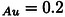 . These profiles show the helical pitch of the Au homopolymer and the more complex structure of the two heterometallic coordination polymers .

Figure 9 shows AFM images on comparable length scales for two heterometallic coordination polymers with x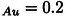 and x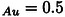 . These strands are about 10 % thicker than poly([Ag(6‐tG)]), but are substantially thinner than poly([Au(6‐tG)]). There are, however, fewer broken strands in both heterometallic coordination polymer samples. The major difference is visible in the height profiles along individual strands (Figure 10); the helicity of poly([Au(6‐tG)]) is clearly visible as a periodic variation in height along the homopolymer, however the height profiles of the heterometallic coordination polymers are more complex. We interpret this in terms of the competition between Ag which leads to left‐handed helices and Au which favours predominantly right‐handed helices. We suggest that the supramolecular structure of the intermediate compositions is different to that of the homopolymers and that this is ultimately responsible for the complex variation in the photoluminescence and circular dichroism with x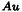 . In a previous report on $\mathrm{Au}{\left(6-\mathrm{tGH}\right)}_{2}$ we have also observed a strong dependence of the photoluminescence spectra on the supramolecular structure.^[34]^

### 2.3 Evidence for Metal Exchange and Equilibration in Mixtures of Homopolymers

The samples discussed so far were prepared by making mixed solutions of metal salts and reacting them with the 6‐tGH ligand. However, we also observed that the polymers themselves are labile to some extent and can exchange metal ions. In the following experiments, samples of poly([Ag(6‐tG)]) and poly([Au(6‐tG)]) homopolymers were prepared first, then mixed and the evolution of their spectroscopic properties with time was followed.

**Figure 11:**
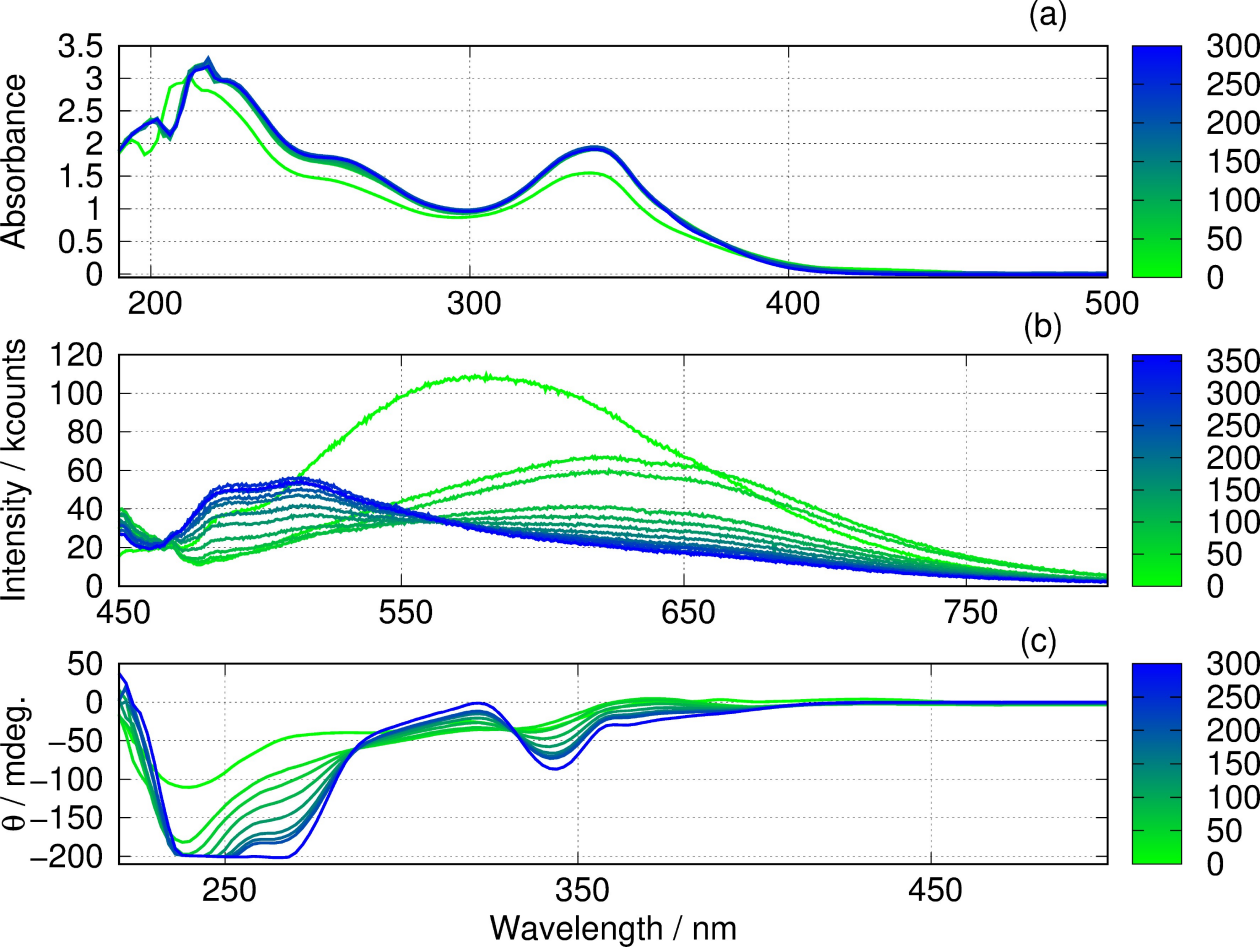
Evolution of optical spectra with time for mixture M3 of the homopolymers. Note that these are mixtures of poly([Au(6‐tG)]) and poly([Ag(6‐tG)]) not mixtures of Ag(I) and Au(I) with 6‐tGH. (a) Absorbance spectra after dilution to 0.1 mM, (b) luminescence spectra (10 mM) and (c) circular dichroism spectra after dilution to 0.2 mM. The colour scale indicates the time after mixing in minutes. In each case, the overall mole fraction of Au was x= 0.5.

Figure 11 shows the change over time in the optical absorption, photoluminescence and circular dichroism spectra for an equimolar mixture of poly([Ag(6‐tG)]) and poly([Au(6‐tG)]). Spectra were recorded in 30 min intervals for 5‐6 h. The absorption spectra (Figure 11a) reach a time‐independent state after 30–60 min. In contrast, the photoluminescence (Figure 11b) and circular dichroism spectra (Figure 11c) continue to evolve for 5‐6 h. The absorption spectra are relatively insensitive to the supramolecular structure and are assigned to transitions localised on the nucleobase.

**Figure 12:**
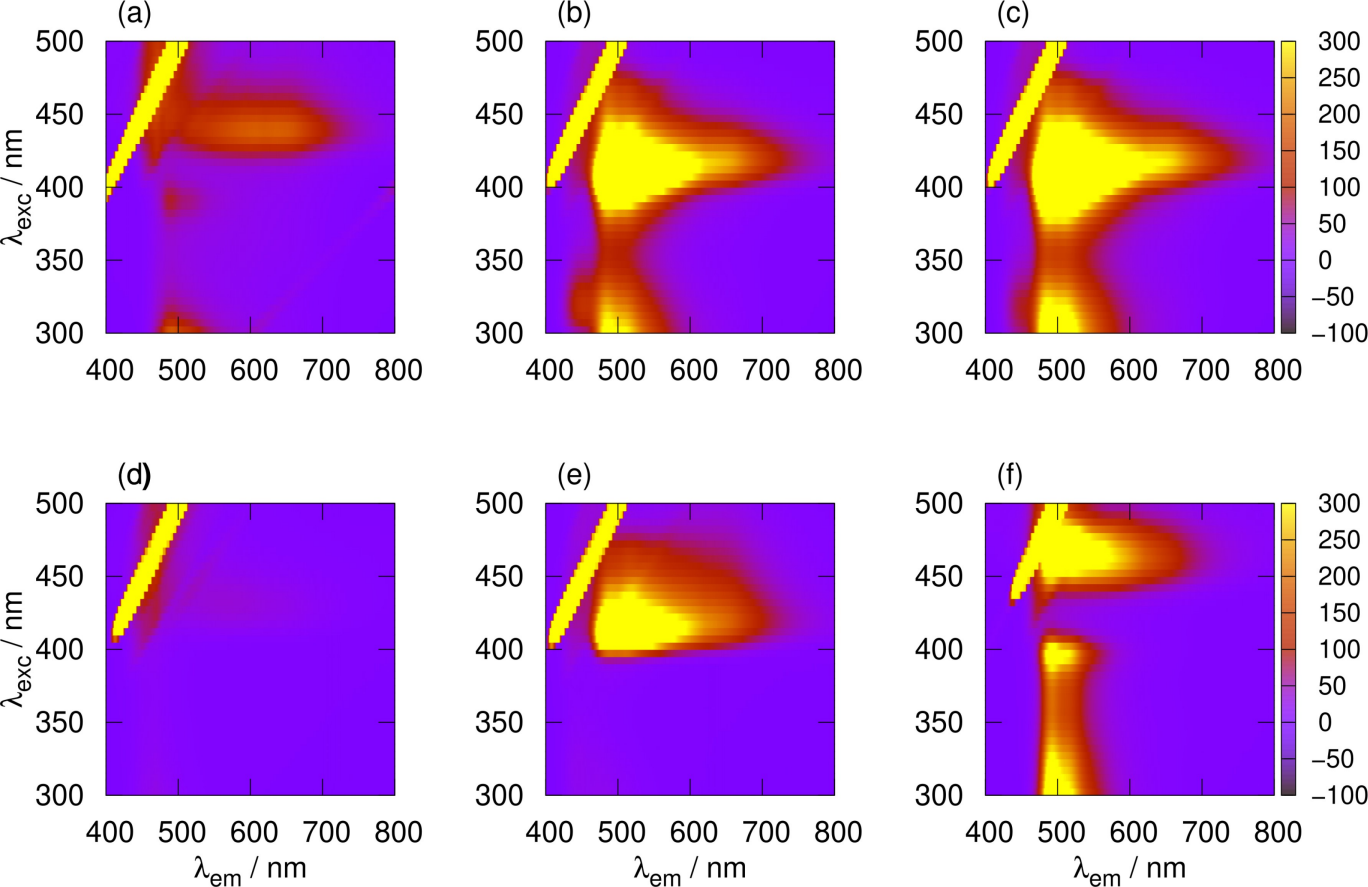
Luminescence excitation‐emission maps for a selection of homopolymers, a 1 : 1 heterometallic coordination polymer poly([${\mathrm{Ag}}_{0.5}{\mathrm{Au}}_{0.5}\left(6-\mathrm{tG}\right)$ ]), and 1 : 1 mixtures of the homopolymers at various times. The total concentration was 10 mM in terms of 6‐tG in each case. (a) M3, 1 : 1 mixture of poly([Ag(6‐tG)]) and poly([Au(6‐tG)]) immediately after mixing at room temperature; (b) M3, 1 : 1 mixture of poly([Ag(6‐tG)]) and poly([Au(6‐tG)]) 5 h after mixing at room temperature; (c) M4, 1 : 1 mixture of poly([Ag(6‐tG)]) and poly([Au(6‐tG)]) 5 h after mixing at 50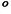 C; (d) homopolymer poly([Ag(6‐tG)]); (e) heterometallic coordination polymer poly($\left[{\mathrm{Ag}}_{0.5}{\mathrm{Au}}_{0.5}\left(6-\mathrm{tG}\right)\right]$ ) and (f) homopolymer poly([Au(6‐tG)]). The y‐axis gives the excitation wavelength, the x‐axis gives the emission wavelength and the colour scale represents the emission intensity.

Initially the photoluminescence spectra are consistent with a mixture of the homopolymers, which both show broad, featureless bands with $550<\lambda_{max}<650$ nm. At longer times the vibronic structure noted in Figure 5 for x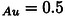 appears. The Stokes shift decreases and the shape of the emission spectrum matches the heterometallic polymer; this indicates that the initial mixture of homopolymers has transformed into a heterometallic coordination polymer by metal exchange. Figure 12 shows the transformation in more detail: after 5 h the maps for the equimolar mixture of poly([Ag(6‐tG)]) and poly([Au(6‐tG)]) (Figure 12b,c) and for the heterometallic coordination polymer poly($\left[{\mathrm{Au}}_{0.5}{\mathrm{Ag}}_{0.5}\left(6-\mathrm{tG}\right)\right]$ ) (Figure 12e) are similar and distinct from both the homopolymers and the equimolar mixture immediately after mixing.

The initial circular dichroism spectra (Figure 11c) show positive components at long wavelengths that resemble the poly([Au(6‐tG)]) homopolymer and a negative band near 230 nm that resembles the poly([Ag(6‐tG)]) homopolymer. With time, the spectrum evolves towards that displayed for x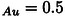 in Figure 7 which is closer in form to the Ag homopolymer which suggests the structure of poly($\left[{\mathrm{Ag}}_{0.5}{\mathrm{Au}}_{0.5}6-\mathrm{tG}\right]$ ) is more like that of poly([Ag(6‐tG)]) than poly([Au(6‐tG)]). Again, the time‐independent spectrum of an equimolar mixture of homopolymers at equilibrium matches the heterometallic coordination polymer spectrum. The optical absorption, photoluminescence and circular dichroism data all show that metal exchange between the homopolymers occurs leading to the formation of a heterometallic coordination polymer.

**Figure 13:**
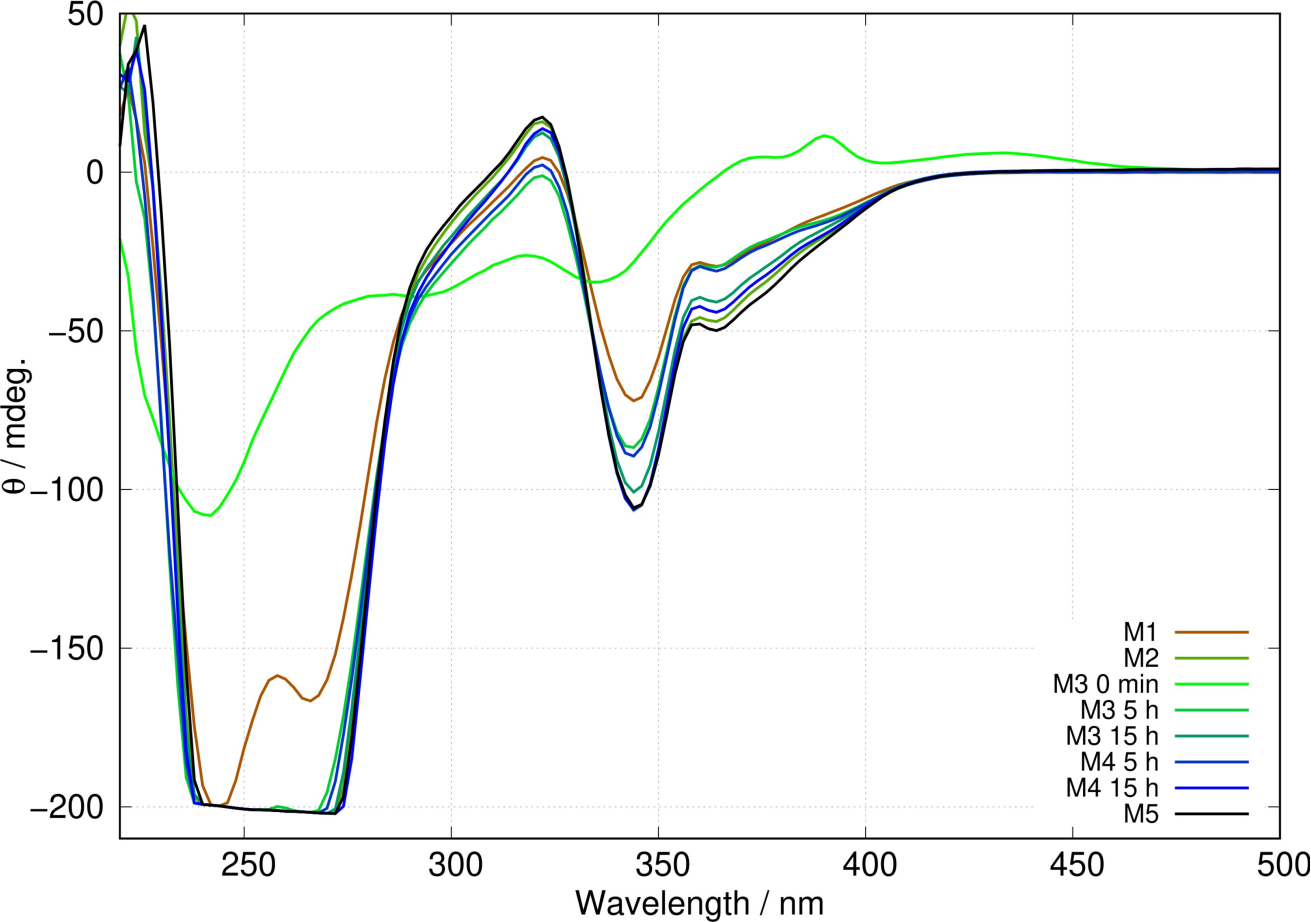
Development of CD spectra of mixtures of Ag and Au homopolymers (M3, M4) compared to heterometallic coordination polymer samples (M1, M2 and M5). The mixtures M1 to M5 are defined in section mixtures. The time after preparation, at which the CD spectrum was recorded, is given in the legend for M3 and M4.

Figure 13 shows the evolution of circular dichroism spectra of various samples of concentration 0.2 mM, all prepared with an overall mole fraction x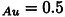 . M1 denotes a sample prepared by addition of 6‐tGH to an equimolar mixture of aqueous ${\mathrm{AgNO}}_{3}$ and Au(I). M2 is similar to M1, except that the solvent was 20 % acetonitrile : 80 % water in which 6‐tGH is very soluble. M3 was prepared by mixing poly([Ag(6‐tG)]) and poly([Au(6‐tG)]) homopolymers in aqueous solution at ambient temperature (about 293 K). M4 was the same as M3, but at 323 K. Finally M5 was prepared by reaction of 6‐tGH with aqueous Au(I) and then addition of ${\mathrm{AgNO}}_{3}$ . The spectra evolve towards that of the poly($\left[{\mathrm{Au}}_{0.5}{\mathrm{Ag}}_{0.5}\left(6-\mathrm{tG}\right)\right]$ ) heterometallic coordination polymer displayed in Figure 7. M3 evolves the slowest, which is expected because this is a mixture of homopolymers rather than small molecules, and is at ambient temperature. However, in all cases the spectra converge to the same endpoint; this is evidence that the final composition of heterometallic coordination polymer is under thermodynamic control.

## 3 Conclusions

The reaction of mixtures of Au(I) and Ag(I) ions with 6‐thioguanosine in aqueous solution produces heterometallic coordination polymers of the form poly($\left[{\mathrm{Au}}_{x}{\mathrm{Ag}}_{1-x}(6-\mathrm{tG})\right]$ ). Rheological data demonstrates that the heterometallic coordination polymers gel as do the corresponding homopolymers. The data indicates shear‐thinning behaviour typical of supramolecular gels. Optical absorption spectra were measured across the composition range from $x_{\mathrm{Au}}=0.0$ to $x_{\mathrm{Au}}=1.0$ . The absorption maximum varies linearly with $x_{\mathrm{Au}}$ from $\lambda_{max}=334.5$ nm for poly([Ag(6‐tG)]) to $\lambda_{max}=369.0$ nm for poly([Au(6‐tG)]). This is the behaviour expected for a random heterometallic coordination polymer rather than a mixture of homopolymers for which the spectra would be represented as a linear combination of the homopolymer spectra weighted according to the metal mole fractions. Microscopic analysis (SEM/EDX, AFM) of the xerogels reveals (i) the heterometallic coordination polymer composition is proportional to the mole fraction of Au(I) in the preparation solution, $x_{\mathrm{Au},\mathrm{Xerogel}}=0.89x_{\mathrm{Au},\mathrm{Soln}}$ and (ii) the heterometallic coordination polymers form long helical structures.

The photoluminescence and circular dichroism spectra are neither simple linear combinations of the homopolymer spectra nor can they be obtained by a linear interpolation between these two limits. This is clearly seen in the emergence of vibronic structure at intermediate compositions x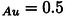 and 0.6 . Instead, these spectra are sensitive to the supramolecular structure of the polymers and show more complex behaviour; photoluminescence spectra of related species are known to be sensitive to metal‐metal interactions^[34]^ and circular dichroism is affected by the helicity of the supramolecular structures. Atomic force microscopy images showed the presence of predominantly right‐hand helices in xerogels of poly([Au(6‐tG)]), which contrasts with poly([Ag(6‐tG)]) which has previously been shown to form left‐hand helices.^[32]^ Intermediate compositions have more complex structures which do not show the same regular helical pitch; we ascribe this to competition between the left and right‐handed preferences of the two limiting cases. The photoluminescence spectra show a broad, featureless band whose intensity increases with Au(I) mole fraction up to about $x_{\mathrm{Au}}=0.3$ . Above $x_{\mathrm{Au}}=0.4$ the spectrum shifts to the blue and shows vibronic structure characteristic of a ligand‐centred transition. At higher mole fractions, the spectrum shifts towards the red again and decreases in intensity. In the circular dichroism spectra, the highest signal intensity is also observed in the intermediate range of mole fractions. Interestingly, the band near 340 nm, which can be assigned to the π system of the nucleobase as perturbed by the metal ion, changes sign between $x_{\mathrm{Au}}=0.0$ and $x_{\mathrm{Au}}=1.0$ in agreement with the AFM observations of the opposite helicity of the Ag and Au homopolymers.

Finally, we studied the lability of the metal‐ligand bonds by preparing homopolymers and then mixing them. Using optical absorption, photoluminescence and circular dichroism spectra to follow the mixtures with time, we observed that they evolved towards the spectra that would have been obtained by preparing a heterometallic coordination polymer directly. We interpret this behaviour as evidence that the composition of the polymer chains is under thermodynamic control and that the metal‐ligand bonds are labile on a timescale of about 5 h at a temperature of about 293 K. This finding is of interest because it suggests that a range of heterometallic coordination polymers could be made by metal exchange. We hypothesise that the present system overcomes previous difficulties with metal exchange in low dimensional systems because of the semi‐rigid nature of the gel.

## Conflict of Interests

The authors declare no conflict of interest.

4

## Supporting information

As a service to our authors and readers, this journal provides supporting information supplied by the authors. Such materials are peer reviewed and may be re‐organized for online delivery, but are not copy‐edited or typeset. Technical support issues arising from supporting information (other than missing files) should be addressed to the authors.

Supporting Information

## Data Availability

The data that support the findings of this study are openly available in https://data.ncl.ac.uk at https://doi.org/10.25405/data.ncl.27652962, reference number 27652962.

## Footnotes

n1 The uncertainty reported is the standard error.
