# Supplementary material for: Metal Exchange in Thioguanosine Coordination Polymers of Gold (I) and Silver (I)
Source: Chemistry. 2025 Feb 21;31(18):e202404318. doi: 10.1002/chem.202404318 (PMC11937882; doi:10.1002/chem.202404318)
Supplement: Supplementary file 1 — Supporting Information [file CHEM-31-e202404318-s001.pdf]

Chemistry–A European Journal

Supporting Information

# Metal Exchange in Thioguanosine Coordination Polymers of Gold (I) and Silver (I)

Chayanan Tangsombun, Liam F. McGarry, Osama El-Zubir, Andrew Houlton,* and Benjamin R. Horrocks*

# Supporting Information for: Metal Exchange in Thioguanosine Coordination Polymers of Gold (I) and Silver (I)

Chayanan Tangsombun, Liam F. McGarry,
Osama El-Zubir, Andrew Houlton* and Benjamin R. Horrocks†

## Contents

**1 Mass Spectrometry** **2**

**2 Protonation model for UV-Vis data** **6**

**3 Rheology of the homopolymers** **7**

**4 Atomic force microscopy** **8**

*andrew.houlton@ncl.ac.uk
†ben.horrocks@ncl.ac.uk

# 1 Mass Spectrometry

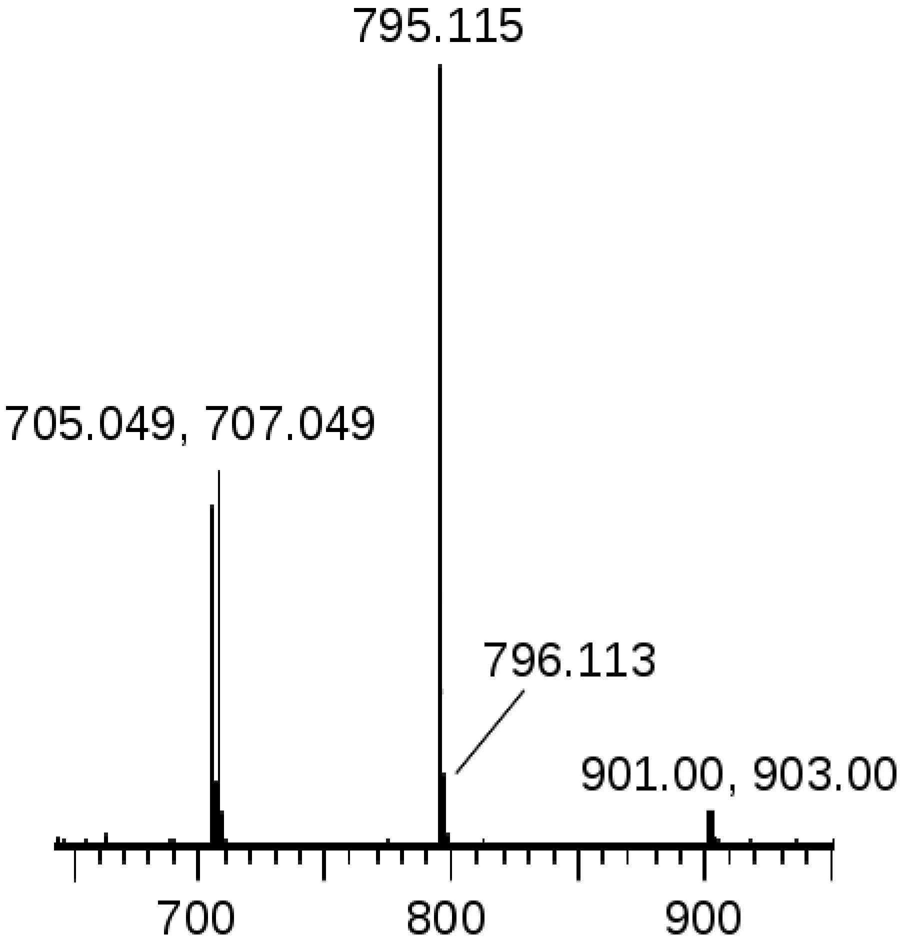


Figure S1: High resolution mass spectrum of poly([$Ag_{0.5}Au_{0.5}$(6−tG)]). Value of m/z for the main fragment peaks (and Ag, Au isotopes) are indicated.

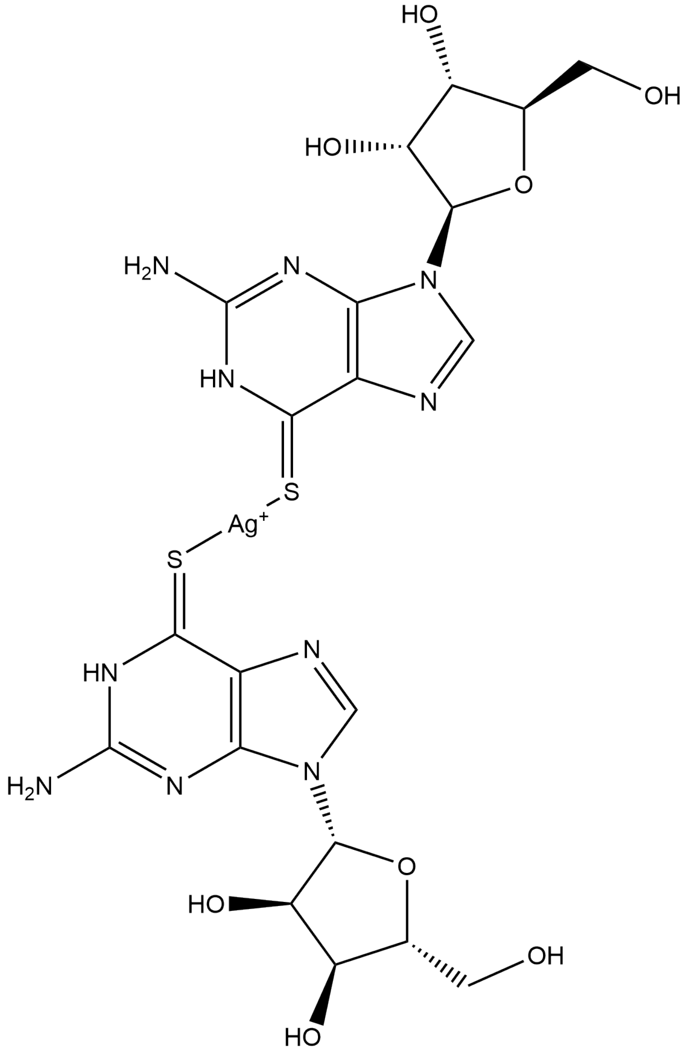


Exact Mass: 705.04

m/z: 707.04 (100.0%), 705.04 (97.3%), 706.05 (21.6%), 708.05 (20.7%), 709.04 (9.2%), 708.04 (7.1%), 706.04 (5.1%), 707.05 (4.2%), 709.05 (4.2%), 710.04 (2.3%)

Figure S2: Structure of $[Ag(6{-}tGH)_2]^+$ and calculated m/z ratios. The values of m/z and the intensities of the corresponding peaks with respect to the most intense peak (assigned a value of 100%) were calculated using ChemDraw 23.1.1 (Revvity Signals Software Inc.).

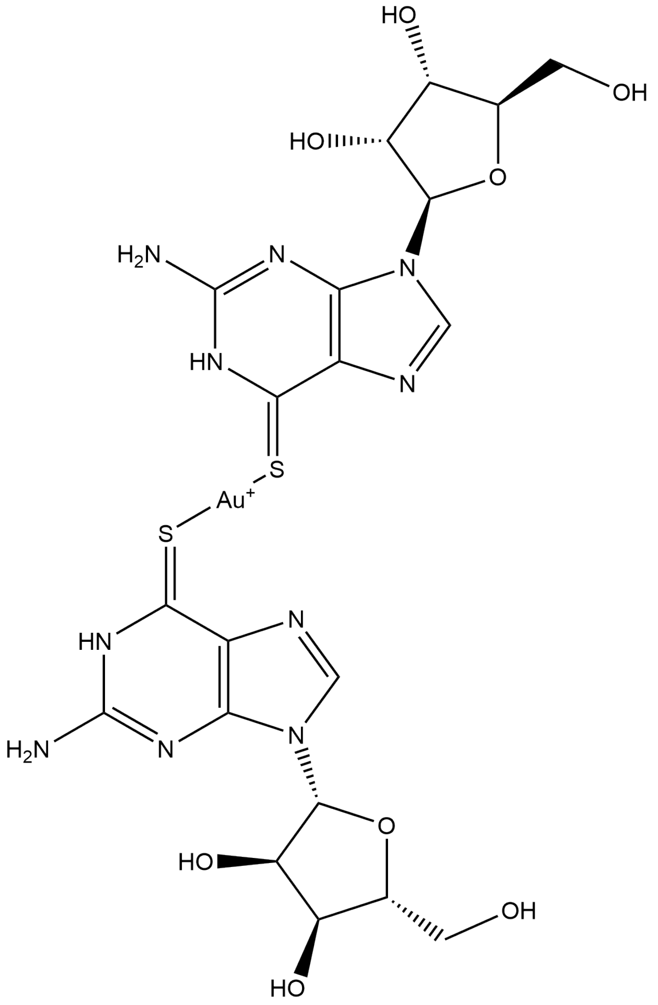


Exact Mass: 795.10

m/z: 795.10 (100.0%), 796.11 (22.2%), 797.10 (9.9%), 796.10 (5.2%), 797.11 (4.4%), 798.10 (2.4%)

Figure S3: Structure of $[Au(6-tGH)_2]^+$ and calculated m/z ratios. The values of m/z and the intensities of the corresponding peaks with respect to the most intense peak (assigned a value of 100%) were calculated using ChemDraw 23.1.1 (Revvity Signals Software Inc.).

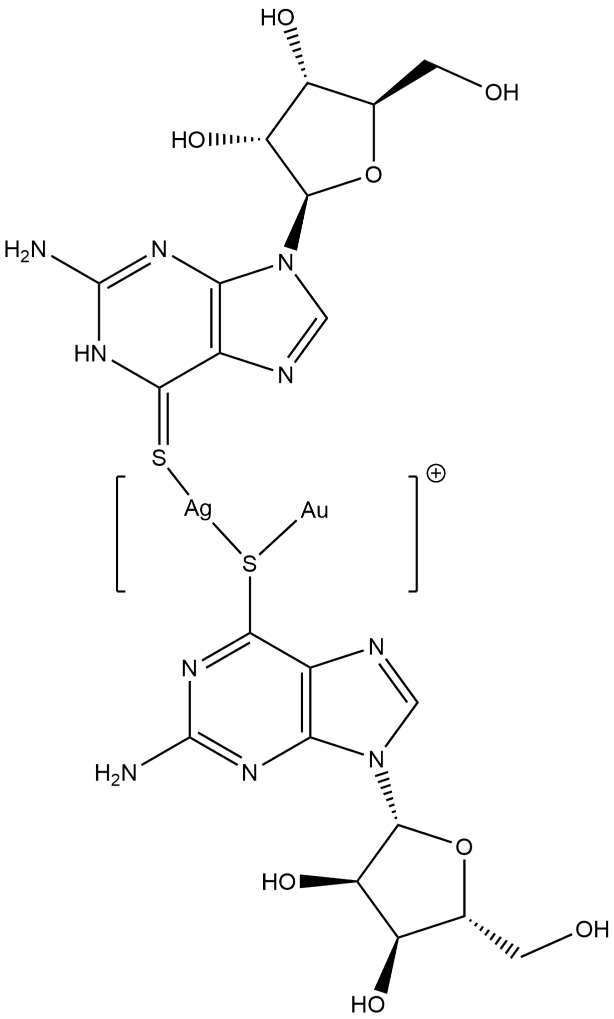


Exact Mass: 901.00
m/z: 903.00 (100.0%), 901.00 (97.0%), 904.00 (26.3%), 902.00 (26.0%), 905.00 (9.7%), 903.01 (3.9%), 905.01 (3.7%), 906.00 (2.1%), 904.01 (1.2%)

Figure S4: Structure of $[AgAu(6-tG)(6-tGH)]^+$ and calculated m/z ratios. The values of m/z and the intensities of the corresponding peaks with respect to the most intense peak (assigned a value of 100%) were calculated using ChemDraw 23.1.1 (Revvity Signals Software Inc.).

# 2 Protonation model for UV-Vis data

$$BH^+ \rightleftharpoons B + H^+ \tag{1}$$

Where $B$ and $BH^+$ are monomer units and protonated monomer units within the polymer. Define the acid dissociation constant, $K_a$, in terms of monomer units, ignoring activity coefficient effects and omitting explicit factors of $c^{\ominus} = 1$ mol kg$^{-1}$.

$$K_a = \frac{[B][H^+]}{[BH^+]} \tag{2}$$

The mole fraction of protonated monomers $BH^+$ is:

$$x_{BH} = \frac{[BH^+]}{[B] + [BH^+]} \tag{3}$$

Denoting the formal concentration of $[H^+]$ added as $F$, the ratio $r$ of added $[H^+]$ to metal is,

$$r = \frac{F}{[B] + [BH^+]} = \frac{F}{M} \ . \tag{4}$$

Where $M$ is the total metal concentration, also equal to $[BH^+] + [B]$. The mole fraction of the protonated form can be calculated as,

$$x_{BH} = \frac{1}{2}\left[1 + r + \frac{K_a}{M} - \sqrt{\left(1 + r + \frac{K_a}{M}\right)^2 - 4r}\ \right] \ . \tag{5}$$

The measured wavelength of maximum absorption, $\lambda_{max}(x_{BH})$, at ratio $r$ is estimated by a linear interpolation,

$$\lambda_{max}(x_{BH}) = x_{BH}\lambda_{max}(BH^+) + (1 - x_{BH})\,\lambda_{max}(B) \tag{6}$$

Equations (5) and (6) define the regression model which was fitted to the optical absorption data for addition of HCl(aq) to poly([Ag(6−tG)]) by the method of least squares.

# 3 Rheology of the homopolymers

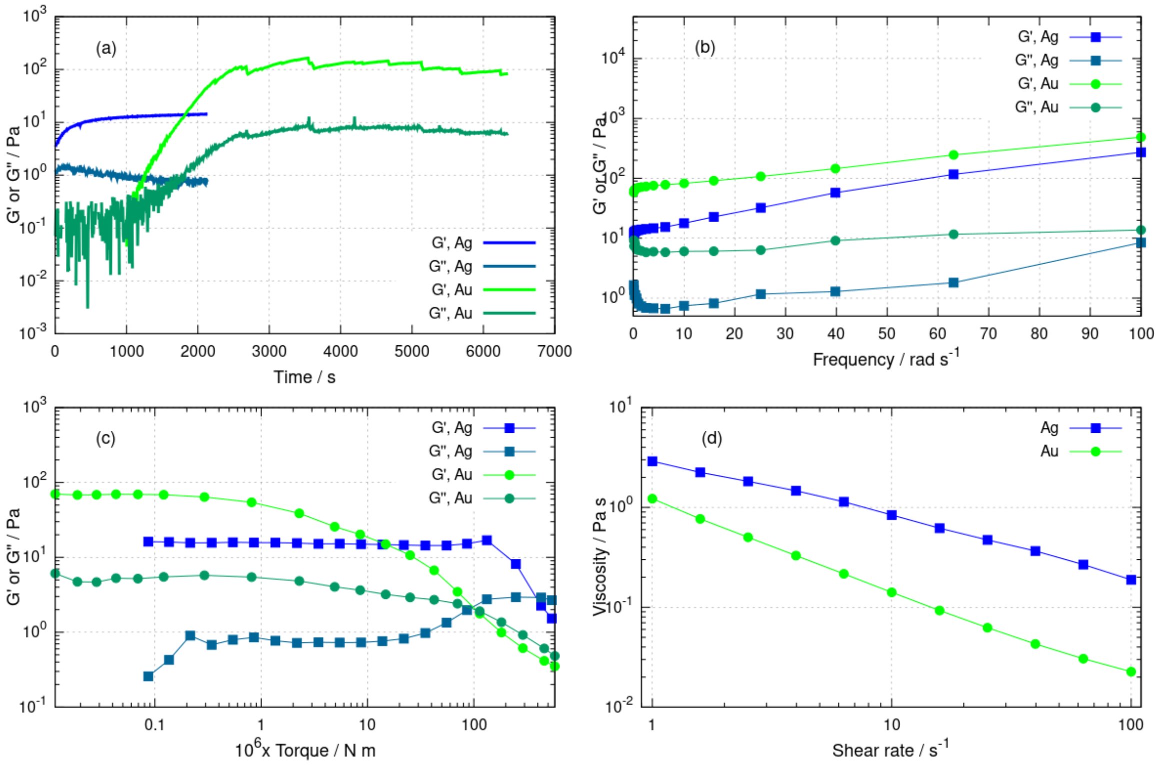


Figure S5: Rheological characterisation of poly([Ag(6−tG)]) and poly([Au(6−tG)]) homopolymers. The concentration of the samples was 10 mM in terms of 6−tG. (a) Time sweep (frequency = 1 Hz); (b) frequency sweep; (c) amplitude (torque) sweep and (d) Viscosity against shear rate. Note that in (d) the viscosity was measured before complete gelation of poly([Au(6−tG)]).

Figure S5 presents the rheological characterisation of the homopolymers: poly([Ag(6−tG)]) and poly([Au(6−tG)]). In Fig. S5a the storage and loss moduli are observed over a period of time following mixing of the ligand and metal. Next, scans of frequency (Fig. S5b) and amplitude (Fig. S5c) were recorded.

Poly([Ag(6−tG)]) forms a gel relatively quickly (minutes) whereas poly([Au(6−tG)]), though forming a stiffer gel ultimately, shows an induction period of about 15 min. The viscosity of the poly([Au(6−tG)]) and poly([Ag(6−tG)]) samples at short times show shear-thinning behaviour (Fig. S5d) consistent with formation of long polymer chains and supramolecular interactions.

## 4 Atomic force microscopy

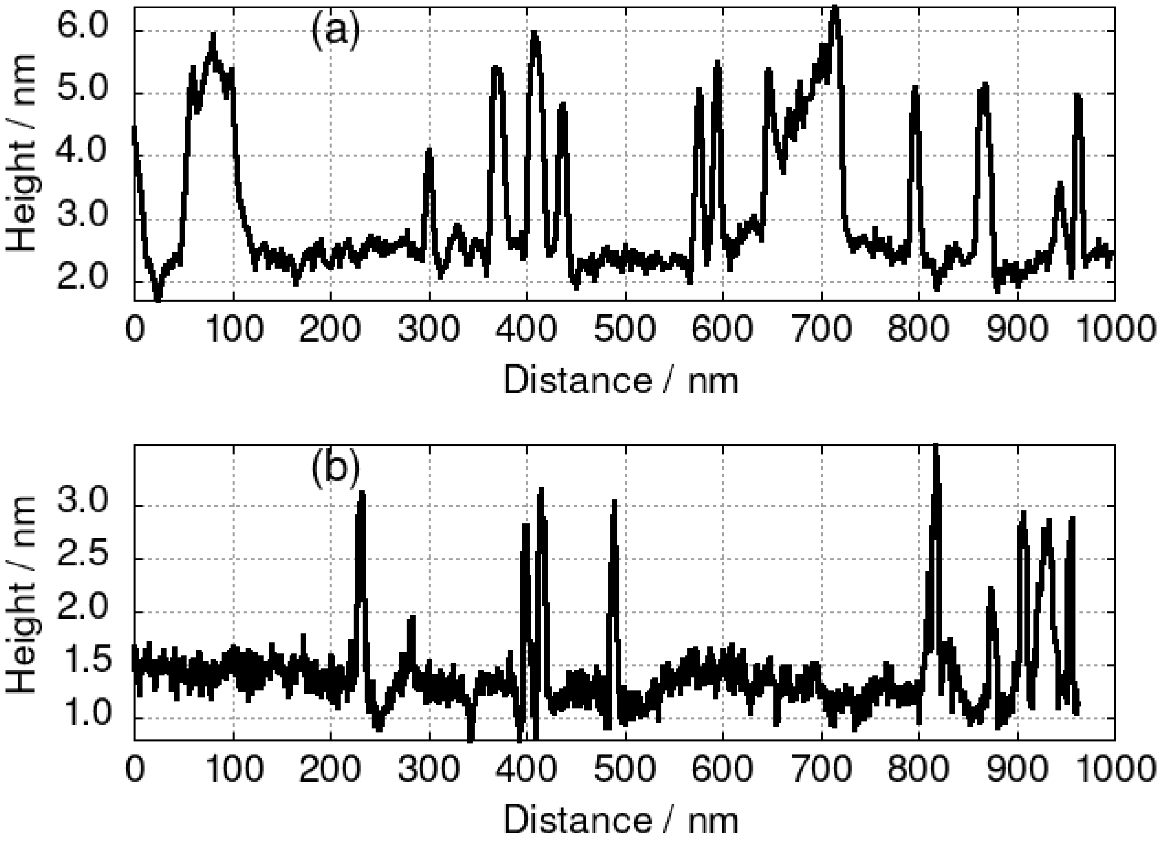


Figure S6: Representative AFM profiles across strands of the (a) poly([Au(6−tG)]) and (b) poly([Ag(6−tG)]) homopolymers. The variation in height indicates the apparent diameter of the strands.

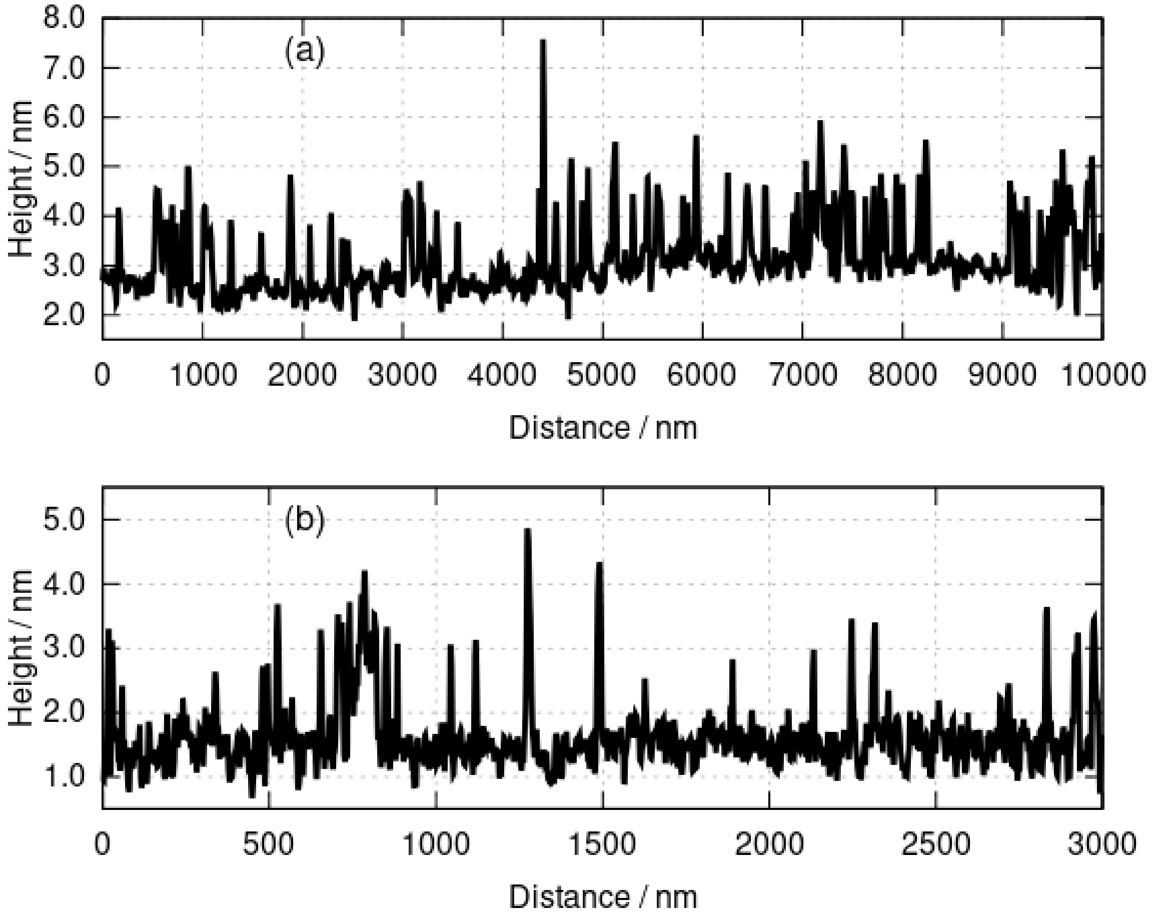


Figure S7: Representative AFM profiles across strands of the (a) poly([$Au_{0.5}Ag_{0.5}$(6−tG)]) and (b) poly([$Au_{0.2}Ag_{0.8}$(6−tG)]) copolymers. The variation in height indicates the apparent diameter of the strands.

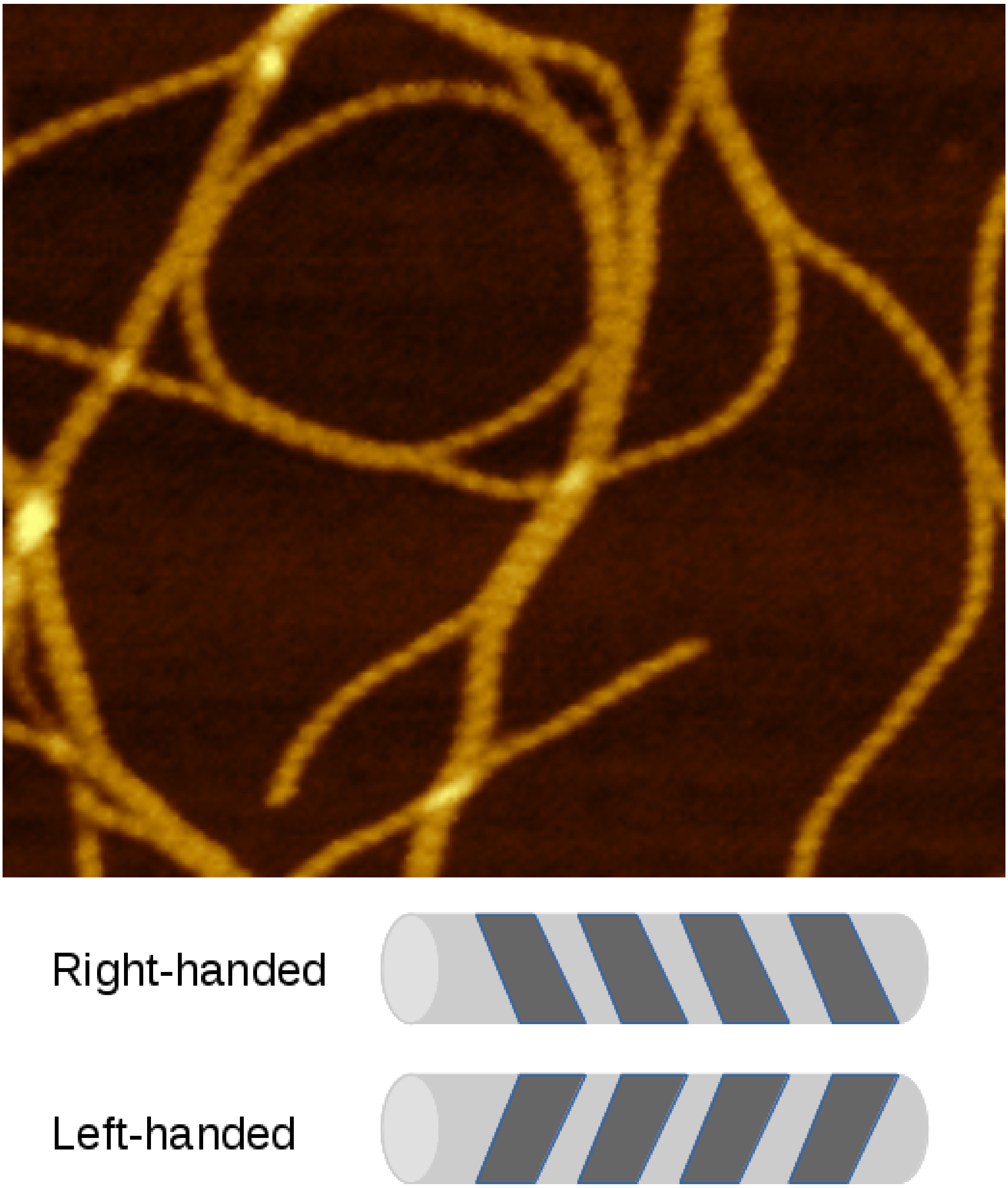


Figure S8: Top: Zoom-in of the AFM image of the Au homopolymer showing the predominantly right-handed nature of the helices. The image size is 400 x 400 nm. Bottom: Schematic diagram illustrating the appearance in AFM of left-handed and right-handed helical fibres lying on a substrate.
